# Rubisco Adaptation Is More Limited by Phylogenetic Constraint Than by Catalytic Trade-off

**DOI:** 10.1093/molbev/msab079

**Published:** 2021-03-19

**Authors:** Jacques W Bouvier, David M Emms, Timothy Rhodes, Jai S Bolton, Amelia Brasnett, Alice Eddershaw, Jochem R Nielsen, Anastasia Unitt, Spencer M Whitney, Steven Kelly

**Affiliations:** 1 Department of Plant Sciences, University of Oxford, Oxford, United Kingdom; 2 Doctoral Training Centre, University of Oxford, Oxford, United Kingdom; 3 Research School of Biology, Australian National University, Canberra, ACT, Australia

**Keywords:** evolution, rubisco, phylogenetic constraint, catalytic constraint, C_4_ photosynthesis

## Abstract

Rubisco assimilates CO_2_ to form the sugars that fuel life on earth. Correlations between rubisco kinetic traits across species have led to the proposition that rubisco adaptation is highly constrained by catalytic trade-offs. However, these analyses did not consider the phylogenetic context of the enzymes that were analyzed. Thus, it is possible that the correlations observed were an artefact of the presence of phylogenetic signal in rubisco kinetics and the phylogenetic relationship between the species that were sampled. Here, we conducted a phylogenetically resolved analysis of rubisco kinetics and show that there is a significant phylogenetic signal in rubisco kinetic traits. We re-evaluated the extent of catalytic trade-offs accounting for this phylogenetic signal and found that all were attenuated. Following phylogenetic correction, the largest catalytic trade-offs were observed between the Michaelis constant for CO_2_ and carboxylase turnover (∼21–37%), and between the Michaelis constants for CO_2_ and O_2_ (∼9–19%), respectively. All other catalytic trade-offs were substantially attenuated such that they were marginal (<9%) or non-significant. This phylogenetically resolved analysis of rubisco kinetic evolution also identified kinetic changes that occur concomitant with the evolution of C_4_ photosynthesis. Finally, we show that phylogenetic constraints have played a larger role than catalytic trade-offs in limiting the evolution of rubisco kinetics. Thus, although there is strong evidence for some catalytic trade-offs, rubisco adaptation has been more limited by phylogenetic constraint than by the combined action of all catalytic trade-offs.

## Introduction

The vast majority of organic carbon on Earth entered the biosphere via the catalytic pocket of rubisco (ribulose-1,5-bisphosphate [RuBP] carboxylase/oxygenase) (Beer et al. 2010). Although there are several metabolic contexts in which this rubisco-mediated reaction can occur, the most important of these in terms of global net primary production is photosynthesis (Andersson and Backlund 2008). Here, rubisco catalyzes the initial step of the Calvin–Benson–Bassham reductive pentose phosphate pathway, catalyzing the fixation of CO_2_ onto the acceptor molecule RuBP to ultimately synthesize sugars. There is a diverse array of rubisco forms found across the tree of life. Plants and some bacteria contain Form I rubisco which is composed of both large (RbcL) and small (RbcS) subunits (Schneider et al. 1992; Tabita et al. 2008), whereas Form II, III, and IV rubisco found in other lineages are comprised of just the large subunit (Tabita et al. 2008; Banda et al. 2020). Although Form I rubisco contains two subunits, only the large subunit is essential for catalysis (Andrews 1988; Lee and Tabita 1990; Whitney et al. 2011), whereas the small subunit has an indirect effect on catalytic properties and activity (Andrews 1988; Lee and Tabita 1990; Lee et al. 1991; Read and Tabita 1992a, 1992b; Spreitzer et al. 2005; Ishikawa et al. 2011; Joshi et al. 2015; Fukayama et al. 2019; Martin-Avila et al. 2020).

Given that rubisco is the entry point for carbon into the global food chain it is perhaps unsurprising that it is the most abundant enzyme on Earth (Ellis 1979) with a global mass of approximately 0.7 gigatons (Bar-On and Milo 2019). However, this abundance is in part due to the inefficiency of rubisco as a catalyst. Specifically, rubisco has a low rate of CO_2_ assimilation (Tcherkez et al. 2006; Savir et al. 2010) and is poorly able to discriminate CO_2_ and O_2_ (Ogren and Bowes 1971) causing it to catalyze both a carboxylation and an oxygenation reaction (Bowes et al. 1971; Chollet 1977; Sharkey 2020). Rubisco-mediated oxygenation of RuBP results in the production of 2-phosphoglycolate, which must then be metabolized to recover carbon and avoid depletion of metabolite pools (Eckardt 2005; Sharwood 2017). In plants, this carbon scavenging process is known as photorespiration, and consumes ATP and reducing power and also liberates ammonia which must be re-assimilated (Peterhansel et al. 2010). Although the oxygenation reaction catalyzed by rubisco is not thought to be deleterious in the anoxic environment prevalent when the enzyme first evolved (Nisbet et al. 2007; Erb and Zarzycki 2018), under current atmospheric conditions it can comprise a quarter of all rubisco reactions in terrestrial plants (Ehleringer et al. 1991). Thus, despite serving a number of beneficial functions (Busch 2020), at its current rate rubisco oxygenation represents a substantial metabolic burden reducing the productivity of plants by up to 50% (Ogren 1984; Bauwe et al. 2012).

Given the high energic cost incurred by the rubisco oxygenation reaction, a number of photoautotrophic organisms have evolved mechanisms to reduce the frequency of its occurrence (Flamholz and Shih 2020). Collectively referred to as CO_2_-concentrating mechanisms, these function to increase the concentration of CO_2_ relative to O_2_ in the vicinity of rubisco and thus increase the relative frequency of carboxylation reactions (Meyer and Griffiths 2013; Flamholz and Shih 2020). These CO_2_-concentrating mechanisms range in complexity from the carboxysome microcompartments in cyanobacteria (Kaplan et al. 1991; Espie and Kimber 2011), to the physical separation of primary CO_2_ assimilation from rubisco-mediated photosynthetic CO_2_ reduction in plants that conduct C_4_ photosynthesis (Hatch 1987; Sage et al. 2012; Edwards 2019). The observation that evolution has resulted in an array of CO_2_-concentrating mechanisms, rather than improve the CO_2_ specificity of rubisco, has led many to question whether altering the kinetics of the enzyme is possible (Ogren 1984; Parry et al. 2007, 2013; Whitney et al. 2011; Sharwood et al. 2016; Orr et al. 2017; Sharwood 2017; Sharkey 2020). This proposition that rubisco specificity cannot be improved was supported by observations that the oxygenase and carboxylase activities of rubisco appear to be tightly linked (Badger and Lorimer 1976; Chollet and Anderson 1976). Subsequently, multiple studies have supported this suggestion by reporting strong antagonistic relationships between rubisco specificity (*S*_C/O_), carboxylase turnover (*k*_catC_), and the Michaelis constant (i.e., an inverse measure of substrate affinity for an enzyme) for CO_2_ (*K*_C_), as well as between *K*_C_ and the Michaelis constant for O_2_ (*K*_O_) (Tcherkez et al. 2006; Savir et al. 2010). Collectively, these studies have led to the hypothesis that severe kinetic trait trade-offs hamstring the inherent efficiency by which the enzyme can catalyze CO_2_ fixation, and that contemporary rubisco are near perfectly adapted within this heavily constrained catalytic landscape (Tcherkez et al. 2006; Savir et al. 2010). However, new evidence has begun to question this paradigm of rubisco evolution. First, recent analyses of the correlative nature of rubisco kinetics has demonstrated that associations between kinetic traits are weakened when a large number of species are considered (Flamholz et al. 2019; Iñiguez et al. 2020). Furthermore, engineering efforts to alter rubisco kinetics have produced enzyme variants that deviate from proposed catalytic trade-offs between *S*_C/O_, *k*_catC_, and *K*_C_ (Wilson et al. 2018; Zhou and Whitney 2019). Finally, an updated examination of rubisco kinetics in the context of other enzymes has shown that it is not as inefficient a catalyst as often assumed (Bathellier et al. 2018). Thus, together these results indicate that rubisco kinetic traits are perhaps not as inextricably linked as originally thought, and suggest that there is scope for increasing the catalytic efficiency of the enzyme as has happened in nature for rubisco in some red algae (Andersson and Backlund 2008; Gunn et al. 2020).

Although the kinetic traits of rubisco appear to be correlated, there are flaws to inferring causality from this correlation. This is because previous analyses that have inferred correlations have assumed that measurements of rubisco kinetic traits in different species are independent (Tcherkez, Farquhar and Andrews 2006; Savir et al. 2010; Flamholz et al. 2019; Iñiguez et al. 2020). However, this assumption has never formally been tested and is unlikely to be true because rubisco in all species are related to each other by descent from a single ancestral gene. This means that rubiscos in closely related species are more similar than rubiscos in species that are more distantly related, a feature which has long been exploited in systematics and evolutionary analyses to serve as an accurate proxy for the phylogenetic relationship between species (Gielly and Taberlet 1994; APG 1998, 2016). As sequence variation determines kinetic variation, closely related enzymes would be expected to also have similar kinetics, with the extent of this similarity being dependent on the underlying tree describing the relationship between species. This phenomenon, which is known as phylogenetic signal, can cause spurious correlations in measured trait values between species unless the structure of the phylogenetic tree is taken into consideration (Felsenstein 1985; Grafen 1989; Pagel and Harvey 1989; Garland 2001). Thus, as previous analyses of rubisco kinetics have not assessed whether a phylogenetic signal exists in rubisco kinetic traits, nor accounted for any phylogenetic signal which may exist, it is possible that the observed catalytic trade-offs inferred from the presence of correlations are, either wholly or in part, an artefact caused by this phylogenetic signal.

Here, we assess the presence of a phylogenetic signal in rubisco kinetic traits to evaluate the extent to which rubisco kinetic evolution is constrained by both phylogenetic effects and catalytic trade-offs. We demonstrate that there is a significant phylogenetic signal in all rubisco kinetic traits. This means that the similarity of kinetic measurements between species varies as a function of their evolutionary distance, and thus kinetic measurements in different species are non-independent. When this phylogenetic signal is correctly accounted for by using phylogenetic least squares regression, we reveal that inferred catalytic trade-offs are weak and that rubisco kinetic traits have been evolving largely independently of each other. Moreover, we find that phylogenetic constraints, most likely resulting from a slow rate of molecular evolution, have constrained rubisco kinetic evolution to a greater extent than catalytic trade-offs. This new insight offers encouragement to efforts which aim to increase yields in food, fiber, and fuel crops by engineering rubisco variants with increased catalytic efficiency.

## Results

### Rubisco Kinetic Data

A data set comprising kinetic measurements for rubisco isolated from different photoautotrophs was obtained from Flamholz et al. (2019). Measurements of specificity (*S*_C/O_) for CO_2_ relative to O_2_ (i.e., the overall carboxylation/oxygenation ratio of rubisco under defined concentrations of CO_2_ and O_2_ gases) in this data set were normalized in order to overcome discrepancies between values determined using an oxygen electrode assay (Parry et al. 1989) and high precision gas-phase-controlled 3H-RuBP-fixation assays (Kane et al. 1994) (see Materials and Methods). To begin, the interrogation of this data was focused on the angiosperms because this was the group with the largest and most complete set of kinetic measurements, and to minimize any impact of long-branch effects (Su and Townsend 2015). It was also restricted to those species with measurements of *S*_C/O_, maximum carboxylase turnover rate per active site (*k*_catC_), and the Michaelis constant (i.e., the substrate concentration at half-saturated catalyzed rate) for both CO_2_ (*K*_C_) and O_2_ (*K*_O_). The Michaelis constant for CO_2_ in 20.95% O_2_ air (*K*_C_^air^) was also inferred as a function of both *K*_C_ and *K*_O_ (see Materials and Methods). Of the 137 angiosperms that satisfied these filtration criteria, 19 also had measurements of the Michaelis constant for RuBP (*K*_RuBP_). From here on, these constants and rates are collectively termed kinetic traits, where *S*_C/O_*, k*_catC_, *K*_C_, and *K*_C_^air^ are referred to as carboxylase-related kinetic traits, and *K*_O_ as the oxygenase-related kinetic trait.

### A Significant Phylogenetic Signal Exists in Rubisco Carboxylase-Related Kinetic Traits in Angiosperms

**Fig. 1.**
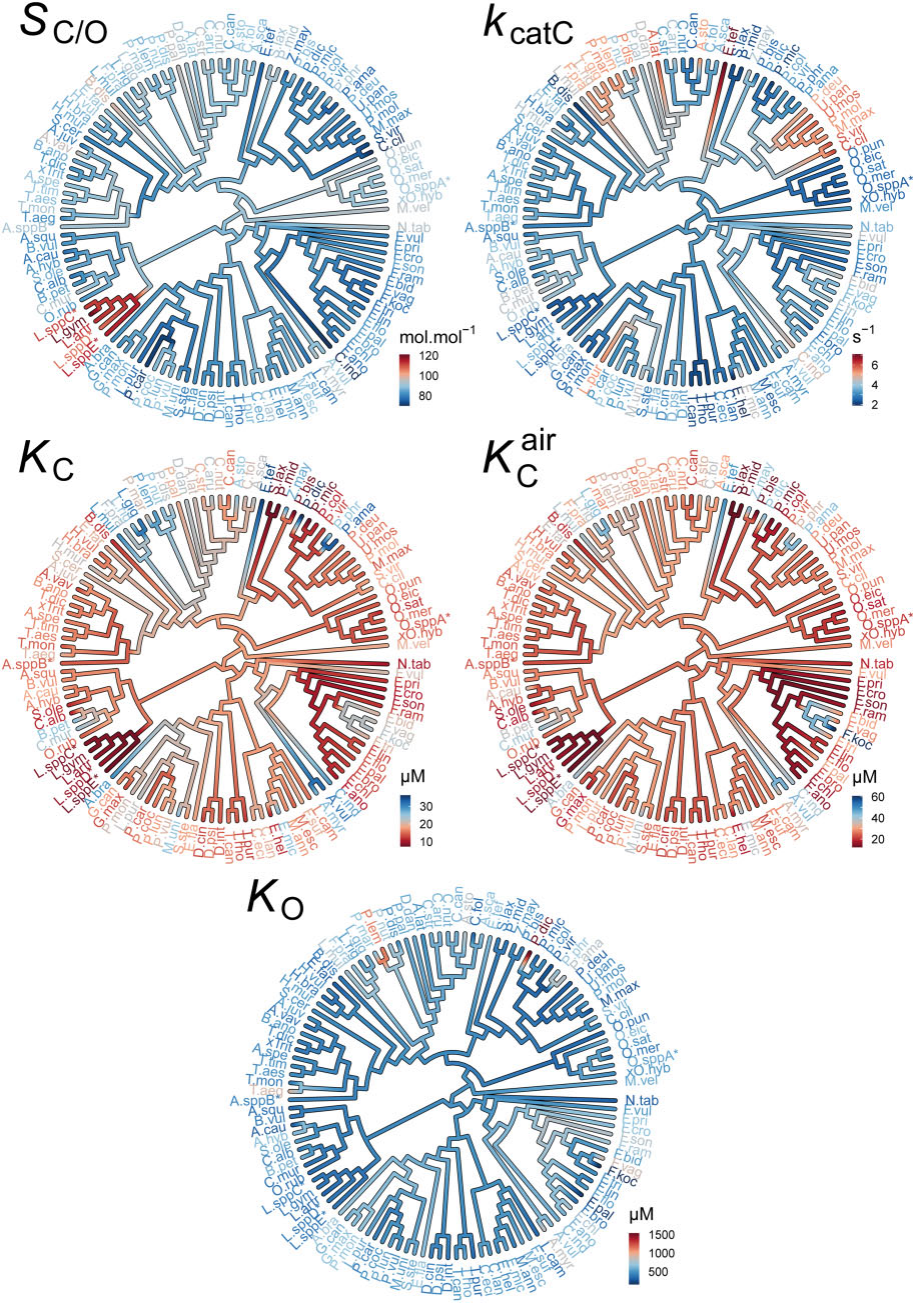
The evolution of rubisco kinetic traits in angiosperms. Phylogenetic tree of angiosperms showing the kinetic trait values in the rubiscos used in this data set and the inferred ancestral kinetic traits for internal branches on the tree. Scale bars for color schemes are presented next to each tree. Species names have been abbreviated for legibility and are provided in full in supplementary file 1, table S4, Supplementary Material online. *S*_C/O_: specificity. *k*_catC_: carboxylase turnover per site. *K*_C_: the Michaelis constant for CO_2_. *K*_C_^air^ the inferred Michaelis constant for CO_2_ in 20.95% O_2_ air_._*K*_O_: the Michaelis constant for O_2_.

Consistent with previous analyses (Flamholz et al. 2019), all kinetic traits were log transformed to ensure they conformed to the distribution assumptions of the statistical analyses herein. To assess whether rubisco in different angiosperms display similar kinetics as a consequence of their phylogenetic relationship, the kinetic traits were analyzed in the context of the phylogenetic tree by which the species are related (fig. 1). Here, all kinetic traits were subject to interrogation for a phylogenetic signal (table 1) except for *K*_RuBP_, which was omitted owing to the limited number of measurements available for this trait. For these analyses, several statistical tools varying in their approach to detection of phylogenetic signal were implemented and the presence or absence of a phylogenetic signal in each trait was judged by the majority result (i.e., the result of ≥3 out of 5 methods tested). Out of the methods utilized, Pagel’s lambda (Pagel 1999) and Blomberg’s *K* and K* (Blomberg et al. 2003) analyze the distribution of trait values in extant species using an explicit Brownian motion model of trait evolution in which the traits evolve stochastically on the underlying phylogenetic tree at a uniform rate and independently among branches. In contrast, Moran’s I (Gittleman and Kot 1990) and Abouheif’s Cmean (Abouheif 1999) do not invoke any specific aspect of evolutionary theory, but instead test for a phylogenetic signal by assessing the correlation of trait values across evolutionary distance on the species tree using the concept of autocorrelation adopted from the field of spatial statistics (Cheverud et al. 1985, 1986). For further discussion of the differences between these phylogenetic signal detection methods, see Münkemüller et al. (2012).

**Table 1.** The Phylogenetic Signal Strength and Associated Significance Level in Rubisco Kinetic Traits in Angiosperms Using Five Different Signal Detection Methods.

Kinetic Trait	*C* mean		*I*		*K*		K*		Lambda	
	*Stat*	*α*	*Stat*	*α*	*Stat*	*α*	*Stat*	*α*	*Stat*	*α*
*S*_C/O_	0.516	0.001	0.425	0.001	0.001	ns	0.001	ns	0.879	0.001
*k*_catC_	0.350	0.001	0.224	0.001	0.003	0.001	0.003	0.001	0.968	0.001
*K*_C_	0.282	0.001	0.248	0.001	0.001	0.01	0.002	0.01	0.902	0.001
*K*_C_^air^	0.234	0.001	0.242	0.001	0.001	0.05	0.001	0.05	0.392	ns
*K*_O_	0.032	ns	−0.070	ns	0	ns	0	ns	0	ns

Note.—Statistics are rounded to three decimal places and significance values are represented as *α* levels, where; *α* = 0.001 if *P *<* *0.001, *α* = 0.01 if 0.001 < *P *<* *0.01, *α* = 0.05 if 0.01 < *P *<* *0.05, and *α* = ns if *P *>* *0.05.

Irrespective of the methodological approach used for inference, a significant phylogenetic signal was observed in all carboxylase-related kinetic traits (*S*_C/O,_*k*_catC,_*K*_C_, and *K*_C_^air^ ) in angiosperms (table 1; fig. 1). However, the strength of this signal varied across the different methods (table 1). In contrast, a phylogenetic signal was not detected for the oxygenase-related kinetic trait *K*_O_ (table 1; fig. 1). These measurements of phylogenetic signal were demonstrated to not suffer from overfitting due to the use of the *rbcL* gene to infer the phylogenetic tree (supplementary file 1, figs. S1 and S2 and table S1, Supplementary Material online). Overall, this means that the similarity in carboxylase-related (but not oxygenase-related) kinetic traits in different angiosperms is dependent on their phylogenetic relationship. Therefore, inferred correlations that assume independence between carboxylase-related kinetic trait values are incorrect, and correlation coefficients computed using such approaches have likely been overestimated (Felsenstein 1985; Grafen 1989; Pagel and Harvey 1989; Gittleman and Kot 1990; Abouheif 1999; Pagel 1999; Garland 2001; Blomberg et al. 2003).

### Significant Changes in Rubisco Kinetics Occur during the Evolution of C_4_ Photosynthesis

**Fig. 2.**
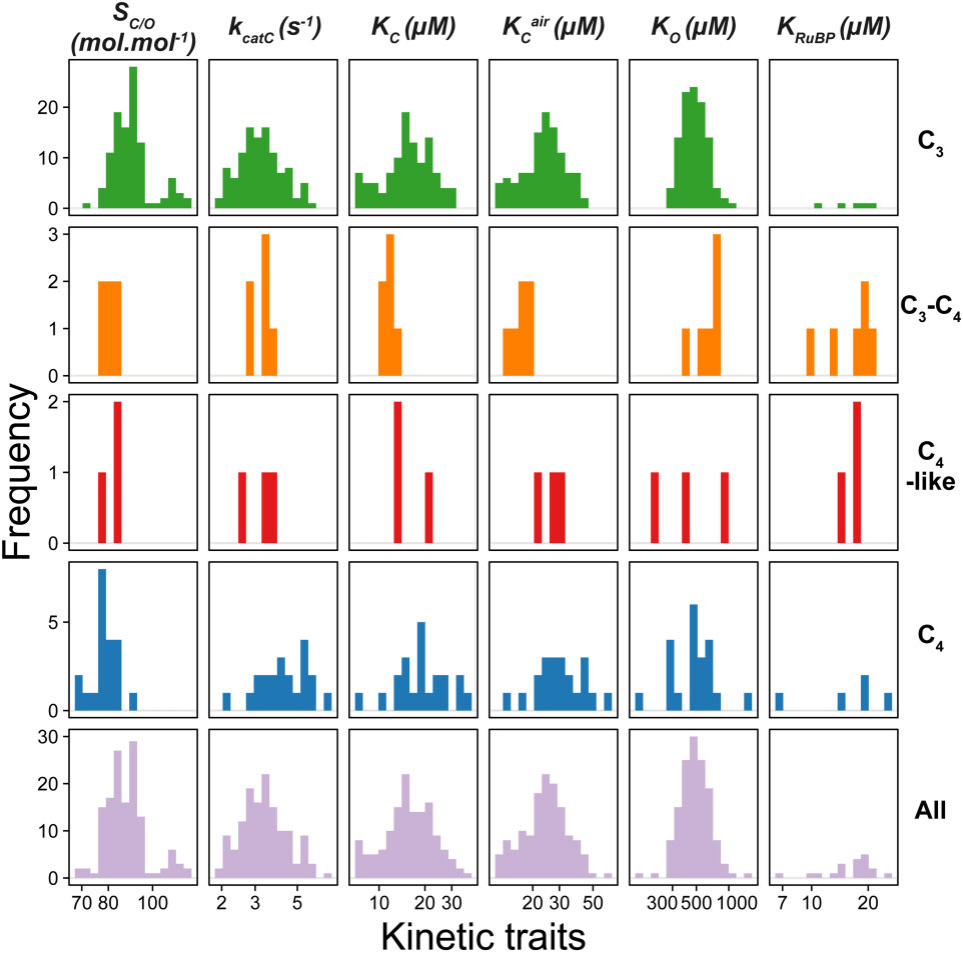
The distributions of values for rubisco kinetic traits in angiosperms. Species are grouped by their photosynthetic type (rows). *S*_C/O_: specificity. *k*_catC_: carboxylase turnover per site. *K*_C_: the Michaelis constant for CO_2_. *K*_C_^air^ the inferred Michaelis constant for CO_2_ in 20.95% O_2_ air_._*K*_O_: the Michaelis constant for O_2_. *K*_RuBP_: the Michaelis constant for ribulose 1,5-bisphosphate. Plants have been classified as those which perform C_3_ photosynthesis (C_3_; *n *=* *107), C_4_ photosynthesis (C_4_; *n *=* *21), C_3_–C_4_ intermediates (C_3_–C_4_; *n *=* *6), C_4_-like (C_4_-like; *n *=* 3*). The *X* axis for all plots is on a log scale, where respective units are shown in column labels. The raw data set used can be found in supplementary file 2, Supplementary Material online.

Inspection of the data identified several dependencies in rubisco kinetic traits between C_3_ and C_4_ plants (fig. 2). Specifically, the mean of the distribution of rubisco *S*_C/O_ values in C_4_ species (mean *S*_C/O_ = 78.7 mol.mol^−1^) was lower than that observed for rubisco in C_3_ species (mean *S*_C/O_ = 89.9 mol.mol^−1^) (fig. 2; *P* < 0.001, *t*-test). Conversely, the mean of the distribution of rubisco *k*_catC_ values was higher in C_4_ species (mean *k*_catC_ = 4.2 s^−1^) than in C_3_ species (mean *k*_catC_ = 3.2 s^−1^) (fig. 2; *P* < 0.001, *t*-test). The means of the distributions of both *K*_C_ and *K*_C_^air^ were also found to be higher in C_4_ species (mean *K*_C_ = 19.0 µM, mean *K*_C_^air^ = 29.9 µM) than in C_3_ species (mean *K*_C_ = 15.4 µM, mean *K*_C_^air^ = 23.6 µM) (fig. 2; *P* < 0.05 and *P* < 0.05, *t*-test, respectively). In contrast, no significant difference was observed in *K*_O_ between C_3_ species (mean *K*_O_ = 481.0 µM) and C_4_ species (mean *K*_O_ = 466.7 µM) (fig. 2; *P* > 0.05, *t*-test). However, variation in *K*_O_ was found to be considerably greater in C_4_ species (95% CI = [379.1, 574.6]) than in C_3_ species (95% CI = [457.1, 506.0]) (*P* < 0.01; Levene test). Although the restricted number of *K*_RuBP_ measurements did not allow statistical differences to be assessed between photosynthetic groups, the distribution of this trait appeared to show higher variability in C_4_ species, similar to that observed for *K*_O_ (fig. 2). Owing to a limited number of kinetic measurements for rubisco in C_3_–C_4_ intermediate and C_4_-like species which respectively represent early and late transition states along the evolutionary continuum from C_3_ to C_4_ photosynthesis, it was not possible to assess changes in rubisco kinetics in these plants relative to the ancestral C_3_ and derived C_4_ photosynthetic types. Nevertheless, trait values of rubisco *S*_C/O_ in both evolutionary intermediate C_3_–C_4_ and C_4_-like states appear to closely resemble the distribution observed in C_4_ species (fig. 2), thus indicating that adaptation of this trait may occur early during the evolution of C_4_ photosynthesis. All of the significant differences between C_3_ and C_4_ plants reported above were robust to correction for phylogenetic signal (supplementary file 1, table S2, Supplementary Material online). Collectively, these data demonstrate that there are adaptations in rubisco kinetics that are associated with the evolution of C_4_ photosynthesis, such that the emergence of the C_4_ carbon concentrating mechanism is accompanied by a decreased specificity and CO_2_ affinity, and an increased carboxylase turnover.

### A Significant Phylogenetic Signal Exists in Rubisco K_O_ in C_3_ Plants

Based on the positions of C_3_–C_4_ intermediate, C_4_-like, and C_4_ species in the phylogenetic tree (supplementary file 1, fig. S1, Supplementary Material online), multiple independent transitions to C_4_ photosynthesis are present in the data set. Furthermore, given that transition to C_4_ photosynthesis is found above to be associated with adaptive changes in rubisco kinetic traits including a reduction in *S*_C/O_, an increase in *k*_catC_, *K*_C_ and *K*_C_^air^, as well as an increased variability in *K*_O_ (fig. 1; supplementary file 1, table S2, Supplementary Material online), it was hypothesized that a failure to account for kinetic differences associated with photosynthetic type may have confounded estimations of the phylogenetic signal. For example, kinetic modifications associated with the evolution of C_4_ photosynthesis may cause larger differences in rubisco kinetics among closely related C_3_ and C_4_ species than expected based on evolutionary distance alone. Similarly, the independent evolution of C_4_ photosynthesis in distantly related plant lineages could also cause evolutionarily distant species to evolve similar kinetic trait values by convergence. To evaluate the extent to which these respective issues may have affected quantification of the phylogenetic signal, the above analyses were repeated using only the C_3_ angiosperm species present (i.e., with C_3_–C_4_ intermediate, C_4_-like, and C_4_ species removed). In general, estimates of the phylogenetic signal in the carboxylase-related kinetic traits in C_3_ species (table 2) agreed with those observed when all angiosperms were considered (table 1). Specifically, a phylogenetic signal of similar strength and significance was observed in *S*_C/O_, *k*_catC_, and *K*_C_ for each of the detection methods across both sets of analyses (tables 1 and 2). In addition, the discrepancies in signal strength between the methods observed for all angiosperms (table 1) were recapitulated in the analysis using only C_3_ species (table 2), thus indicating that these differences are not caused by a failure to control for photosynthetic type, but instead more likely represent distinctions in the assumptions and aspects of the phylogenetic signal measured by each test (Hardy and Pavoine 2012; Münkemüller et al. 2012). In summary, there is a statistically significant phylogenetic signal in rubisco specificity, carboxylase turnover, and the Michaelis constant for CO_2_ in angiosperms that is independent of photosynthetic type.

**Table 2.** The Phylogenetic Signal Strength and Associated Significance Level in Rubisco Kinetic Traits in C_3_ Species Using Five Different Signal Detection Methods.

Kinetic Trait	*C* mean		*I*		*K*		K*		Lambda	
	*Stat*	*α*	*Stat*	*α*	*Stat*	*α*	*Stat*	*α*	*Stat*	*α*
***S*_C/O_**	0.533	0.001	0.453	0.001	0	ns	0.001	ns	0.814	0.001
***k*_catC_**	0.387	0.001	0.234	0.001	0.002	0.01	0.002	0.01	0.913	0.001
***K*_C_**	0.449	0.001	0.341	0.001	0.001	0.05	0.001	0.05	0.948	0.001
***K*_C_^air^**	0.398	0.001	0.317	0.001	0.001	0.05	0.002	0.01	0.947	0.001
***K*_O_**	0.279	0.001	0.167	0.01	0	ns	0	ns	0.743	0.001

Note.—Statistics are rounded to three decimal places, and significance values are represented as *α* levels, where *α* = 0.001 if *P *<* *0.001, *α* = 0.01 if 0.001 < *P *<* *0.01, *α* = 0.05 if 0.01 < *P *<* *0.05, and *α* = ns if *P *>* *0.05.

In contrast to the analysis of all angiosperms (table 1), a significant phylogenetic signal was observed in *K*_O_ when only C_3_ angiosperms were considered (table 2). Thus, both the oxygenase-related and carboxylase-related traits of rubisco have evolved in a tree-like manner in C_3_ angiosperms. Furthermore, unlike the other carboxylase-related kinetic traits, the phylogenetic signal in *K*_C_^air^ was found to increase in strength when the analysis is restricted to C_3_ angiosperms. This result is a corollary of the fact that *K*_C_^air^ is computed here from both *K*_C_ and *K*_O_. Thus, all kinetic traits of rubisco have a significant phylogenetic signal in C_3_ angiosperms.

### Correlations between Kinetic Traits Are Weak in Angiosperms and Further Relaxed after Correcting for a Phylogenetic Signal

**Fig. 3.**
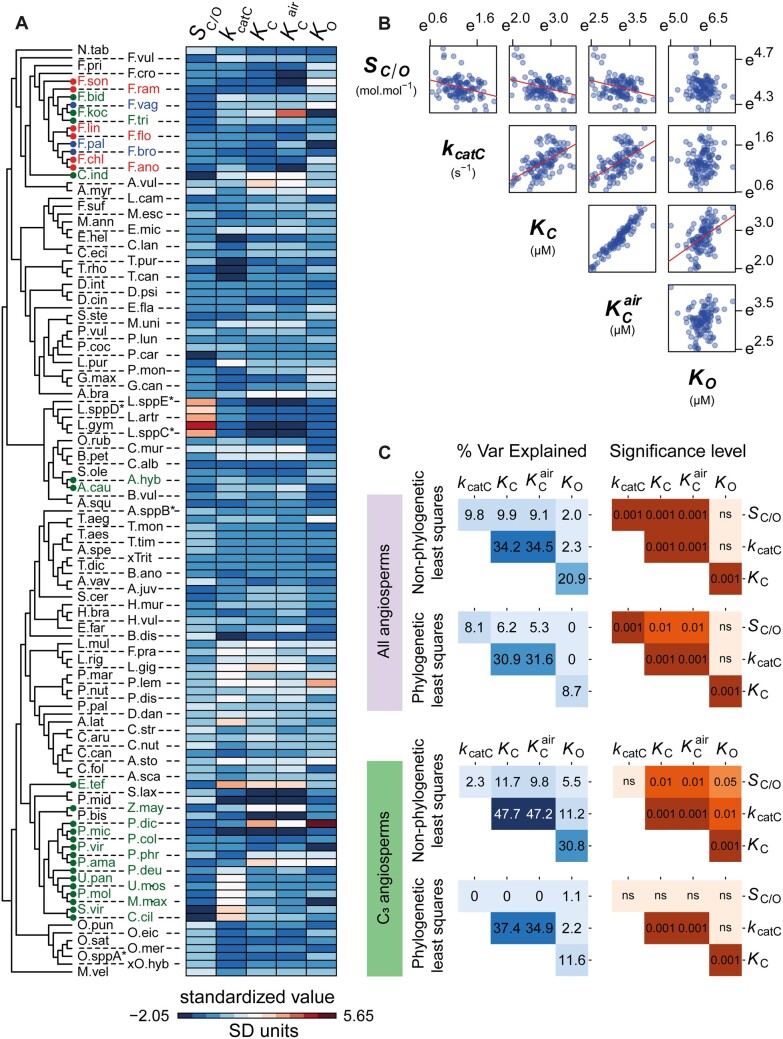
The correlations between rubisco kinetic traits in angiosperms. (*A*) Heatmap depicting the variation in kinetic traits across the species used in this study (± SD away from each respective kinetic trait mean). Species labels on the tree are color coded by photosynthetic type (C_3_: black, C_3_–C_4_ intermediates: red, C_4_-like: blue, and C_4_: green), and have been abbreviated for legibility (for full names refer to supplementary file 1, table S4, Supplementary Material online). (*B*) The relationships between all pairwise combinations of log transformed rubisco kinetic traits. (*C*) Pairwise correlation coefficients (percent variance explained) and associated *P*-values between rubisco kinetic traits assessed using non-phylogenetic least squares regression models or phylogenetic least squares regression models. Phylogenetic and non-phylogenetic least squares regressions were fit to both the complete set of angiosperms in the data set and the subset which perform C_3_ photosynthesis. Significance values are represented as *α* levels, where; *α* = 0.001 if *P *<* *0.001, *α* = 0.01 if 0.001 < *P *<* *0.01, *α* = 0.05 if 0.01 < *P *<* *0.05, and *α* = ns if *P *>* *0.05.

Given the finding that rubisco kinetic traits exhibit a significant phylogenetic signal (table 1; fig. 2), it is possible that previously reported correlations between rubisco kinetic traits (Tcherkez et al. 2006; Savir et al. 2010; Flamholz et al. 2019; Iñiguez et al. 2020) are an artefact of this signal. This is because prior analyses which did not account for the inherent phylogenetic structure (and non-independence) of this data (fig. 3*A*) may have overestimated correlation coefficients due to this underlying structure. Thus, in order to evaluate the extent to which phylogenetic signal may have influenced previous results (Tcherkez et al. 2006; Savir et al. 2010; Flamholz et al. 2019; Iñiguez et al. 2020), the correlations observed in the kinetic trait data using both phylogenetic and non-phylogenetic regression methods were compared (fig. 3*B* and *C*).

Using a standard non-phylogenetic approach, the relationships between kinetic traits of rubisco were consistent in both linear and least squares regression models (supplementary file 1, fig. S3*A* and *B*, Supplementary Material online). The direction of the power–law relationships observed (fig. 3*B*) match those previously reported (Flamholz et al. 2019). Specifically, significant positive correlations were found between *k*_catC_ and both *K*_C_ and *K*_C_^air^ (fig. 3*B* and *C*). A significant positive correlation was also observed between the respective Michaelis constants for both CO_2_ and O_2_ substrates, *K*_C_ and *K*_O_ (fig. 3*B* and *C*). In addition, significant inverse power–law correlations were observed between *S*_C/O_ and all other carboxylase-related kinetic traits, including *k*_catC_, *K*_C_, and *K*_C_^air^ (fig. 3*B* and *C*). In contrast, *K*_O_ did not co-vary with either *S*_C/O_ or *k*_catC_ (fig. 3*B* and *C*), whereas *K*_RuBP_ did not appear to be tightly linked to any kinetic trait from the limited number of observations that are available (supplementary file 1, fig. S3*A* and *B*, Supplementary Material online). Thus, across angiosperms, all pairwise relationships between the carboxylase-related kinetic traits *S*_C/O_, *k*_catC_, and either *K*_C_ or *K*_C_^air^ were significant, whereas the oxygenase-related trait *K*_O_ was only correlated with *K*_C_.

Although kinetic trade-offs inferred using non-phylogenetic methods were concordant in direction with those previously described (Flamholz et al. 2019), they were substantially reduced in magnitude when the analysis was focused solely on the angiosperms. This reduction in magnitude of correlation when taxonomic groups are removed is strongly indicative of phylogenetic signal in the data set and is analyzed in further detail in a subsequent results section. Within angiosperms, the strength of the correlation between *S*_C/O_ and *K*_C_ (9.9% variance explained; fig. 3*C*) is attenuated by 77% when compared with that previously reported using a larger range of rubisco variants (43.6% variance explained; Flamholz et al. 2019). Moreover, a 69% reduction was found in the dependency between *S*_C/O_ and *k*_catC_ in angiosperms (9.8% variance explained; fig. 3*C*) in comparison to that reported based on the larger range of species (31.4% variance explained; Flamholz et al. 2019), with the antagonistic correlation observed between *K*_C_ and *K*_O_ (20.9% variance explained; fig. 3*C*) also weakened by 33% relative to previous reports (31.4% variance explained; Flamholz et al. 2019). In contrast, the dependency between *K*_C_ and *k*_catC_ was 49% stronger when only angiosperms are assessed, increasing from 23.0% (Flamholz et al. 2019) to 34.2% in this study (fig. 3C). Therefore overall, even in the absence of correctly accounting for the phylogenetic relationship between rubisco, the apparent catalytic trade-offs observed in angiosperms are weaker than previously thought (Flamholz et al. 2019; Iñiguez et al. 2020).

Given that a significant phylogenetic signal is present in rubisco kinetic traits in angiosperms (tables 1 and 2), a phylogenetic generalized least squares regression analysis (Felsenstein 1985) was conducted to estimate the magnitude of the catalytic trade-offs when accounting for the inherent structure of the data. In comparison to phylogeny-unaware correlations, the phylogenetic regression resulted in a reduction in the majority of kinetic trade-offs (fig. 3*C*). The largest reduction observed was for the correlation between the Michaelis constants *K*_C_ and *K*_O._ Here, the correlation was reduced by 58% (variance explained = 8.7%) relative to methods which do not correctly account for the non-independence of these measurements (variance explained = 20.9%; fig. 3*C*). An analogous weak correlation was also observed when the phylogenetic analyses were limited to C_3_ species (variance explained = 11.6%; fig. 3*C*). Thus, changes in rubisco *K*_C_ have occurred largely independent of any change on *K*_O_ during the diversification of the angiosperms.

Phylogenetic correction also resulted in less substantial reductions in the correlation between *S*_C/O_ and each of the other carboxylase-related traits (fig. 3*C*). Here the dependency between *S*_C/O_ and *k*_catC_ was reduced by 18% from 9.8% to 8.1%, whereas the dependency between *S*_C/O_ and both *K*_C_ and *K*_C_^air^ was reduced by 37% and 42% from 9.9% to 6.2%, and from 9.1% to 5.3%, respectively (fig. 3*C*). Furthermore, these correlations were not significant when considering only C_3_ species (fig. 3*C*). Thus, during the evolution of rubisco in angiosperms, changes to specificity have occurred with little or no effect on other carboxylase-related kinetic traits, and *vice versa*.

In contrast, the strength of the correlation between *k*_catC_ and either *K*_C_ or *K*_C_^air^ was robust to phylogenetic correction. Specifically, the dependency between *k*_catC_ and *K*_C_ only decreased by 10% from 34.2% to 30.9%, and the dependency between *k*_catC_ and *K*_C_^air^ decreased by only 8% from 34.5% to 31.6% (fig. 3*C*). Furthermore, the phylogenetically corrected correlations between these kinetic traits were of a similar magnitude when only C_3_ species were considered (37.4% and 34.9%, respectively; fig. 3*C*). Thus, as rubisco kinetics have evolved in angiosperms, there has been a trade-off between CO_2_ affinity and carboxylase turnover such that any change in one kinetic trait caused a partial change in the other, though with little impact on any further rubisco kinetic traits.

### The Evolution of Rubisco Kinetics Is More Limited by Phylogenetic Constraints Than by Catalytic Trade-Offs in Angiosperms

**Fig. 4.**
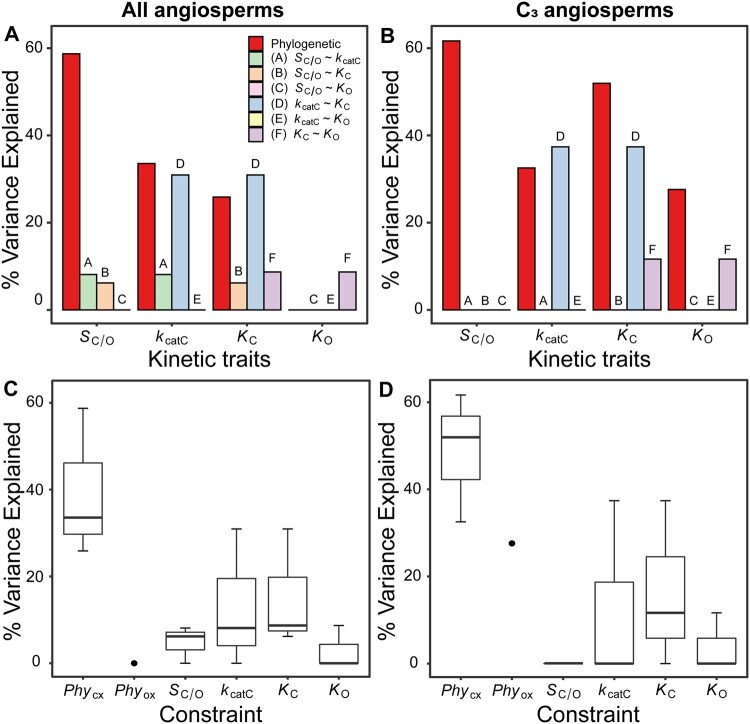
The constraints on rubisco kinetic adaptation in angiosperms. (*A*) The variation (%) in rubisco kinetic traits across angiosperms that can be explained by phylogenetic constraint and each catalytic trade-off. (*B*) As in (A) but for C_3_ angiosperms only. (*C*) Boxplot of all variation explained in each kinetic trait by kinetic trait correlations in comparison to variation explained by phylogeny in angiosperms. The phylogenetic constraints on the carboxylase-related traits *Phy*_CX_ (includes *Phy_Sc/o_*, *Phy_Kcatc_*, and *Phy_Kc_*) and phylogenetic constraints on the oxygenase-related trait *Phy*_ox_ (includes *Phy_Ko_* only) are presented separately. (*D*) As in (C) but for C_3_ angiosperms only.

As rubisco kinetic traits contain a phylogenetic signal in angiosperms (tables 1 and 2), we sought to determine the extent to which the phylogenetic signal was caused by phylogenetic constraint. Here, phylogenetic constraint is considered to comprise all constraints which are embedded within the structure of the phylogenetic tree, that are independent of the kinetic constraints, and collectively act to impede the adaptive evolution of rubisco kinetics. For example, such phylogenetic constraints include processes which lead to neutral evolution (Felsenstein 1985) or evolutionary stasis (Prinzing et al. 2001; Ackerly 2004; Cavender-Bares et al. 2004; Moles et al. 2005; Swenson and Enquist 2007) of the trait in question (Ives 2019; Nevo et al. 2020). In order to assess the relative strength of such phylogenetic constraints on rubisco kinetics, the variance in kinetic traits partitioned between phylogenetic effects (i.e., the explanatory power of the phylogenetic tree in the goodness-of-fit model and a measure of phylogenetic constraint) and non-phylogenetic effects (i.e., the remaining explanatory power of the regression model, accounted for by the sum of all other constraints such as random effects and all kinetic trait trade-offs) was quantified. This analysis revealed that phylogenetic constraints explained a significant proportion of the variation in either the carboxylase-related kinetic traits across all angiosperms (fig. 4*A*), or in all kinetic traits across C_3_ angiosperms (fig. 4*B*). With one exception (i.e., the phylogenetic constraint on *K_O_* in the larger species data set) the magnitude of variation explained by phylogenetic constraints was similar or larger to the variation explained by the strongest trade-off observed between *k*_catC_ and *K*_C_ (fig. 4*C* and *D*). Consequently, in angiosperms, the cumulative variance explained by phylogenetic constraints across all rubisco kinetic traits (29.5%) was larger than the cumulative variance for all catalytic trade-offs combined (9.0%). This effect was more pronounced for C_3_ angiosperms (cumulative variance for phylogenetic constraints = 43.4%, cumulative variance for catalytic trade-offs = 8.2%). Thus, during the radiation of angiosperms phylogenetic constraints have restricted the evolution of rubisco kinetics to a greater extent than all catalytic trade-offs combined.

### Phylogenetic Signal, Weak Kinetic Trait Correlations, and Strong Phylogenetic Constraint Are Features of Rubisco Evolution in All Photosynthetic Organisms

Given the presence of phylogenetic signal and the impact of phylogenetic constraint on the evolution of rubisco kinetics in angiosperms, we sought to determine whether these findings were a unique feature of angiosperm rubisco or whether they were a more general phenomenon across the tree of life. To achieve this, the data set was expanded to include all species for which both kinetic measurements and an *rbcL* gene sequence were available. Analogous to the analysis of angiosperms, a strong and statistically significant phylogenetic signal was observed in *S*_C/O_, *k*_catC_, *K*_C_, and *K*_C_^air^, but not in *K*_O_ across all photosynthetic organisms (table 3). Similarly, a significant phylogenetic signal was also observed for *K*_O_ when C_3_–C_4_, C_4_-like, and C_4_ angiosperms were omitted to control for the dependency in kinetic trait measurements on the tree associated with the convergent transition to C_4_ photosynthesis in angiosperms (supplementary file 1, table S3, Supplementary Material online). Thus, there is a significant phylogenetic signal in rubisco kinetic traits in all photosynthetic organisms.

**Table 3.** The Phylogenetic Signal Strength and Associated Significance Level in Rubisco Kinetic Traits across All Studied Photosynthetic Organisms Using Five Different Signal Detection Methods.

Kinetic Trait	*C* mean		*I*		*K*		K*		Lambda	
	*Stat*	*α*	*Stat*	*α*	*Stat*	*α*	*Stat*	*α*	*Stat*	*α*
*S*_C/O_	0.712	0.001	0.229	0.001	0.005	0.001	0.002	0.001	1.005	0.001
*k*_catC_	0.538	0.001	0.29	0.001	0.002	0.001	0.001	0.001	0.975	0.001
*K*_C_	0.705	0.001	0.277	0.001	0.002	0.001	0.001	0.001	0.948	0.001
*K*_C_^air^	0.609	0.001	0.299	0.001	0.001	0.001	0.001	0.01	0.924	0.001
*K*_O_	0.170	0.001	0.004	ns	0	ns	0	ns	0.603	0.001

Note.—Statistics are rounded to three decimal places and significance values are represented as *α* levels, where *α* = 0.001 if *P *<* *0.001, *α* = 0.01 if 0.001 < *P *<* *0.01, *α* = 0.05 if 0.01 < *P *<* *0.05, and *α* = ns if *P *>* *0.05.

**Fig. 5.**
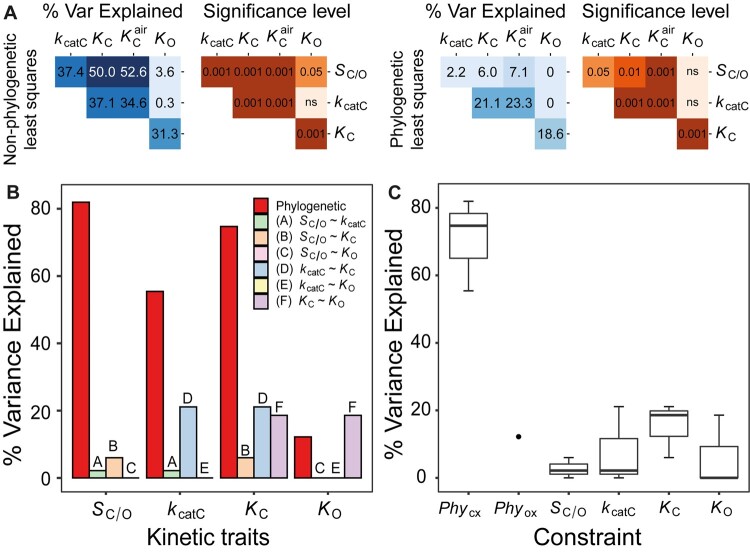
Kinetic and phylogenetic constraints on rubisco adaptation across all photosynthetic organisms. (*A*) Pairwise correlation coefficients (percent variance explained) and associated *P*-values between different rubisco kinetic traits assessed using non-phylogenetic least squares regression models or phylogenetic least squares regression models. Significance values are represented as *α* levels, where *α*  =  0.001 if *P *<* *0.001, *α*  =  0.01 if 0.001 < *P *<* *0.01, *α*  =  0.05 if 0.01 < *p *<* *0.05, and *α* = ns if *P *>* *0.05. (*B*) The variation (%) in rubisco kinetic traits across all photosynthetic organisms that can be explained by phylogenetic constraint and each catalytic trade-off. (*C*) Boxplot of all variation explained in each kinetic trait by kinetic trait correlations in comparison to variation explained by phylogeny in all photosynthetic organisms. The phylogenetic constraints on the carboxylase-related traits *Phy*_CX_ (includes *Phy_Sc/o_*, *Phy_Kcatc_*, and *Phy_Kc_*) and phylogenetic constraints on the oxygenase-related trait *Phy*_ox_ (includes *Phy_Ko_* only) are presented separately.

Analogous to the above analyses, accounting for the phylogenetic tree (supplementary file 1, fig. S4, Supplementary Material online) caused a substantial attenuation in the kinetic trait correlations in all species (fig. 5*A*;supplementary file 1 and figs. S3*C*, S5, and S6, Supplementary Material online). Specifically, when correcting for the phylogenetic signal in kinetic traits, a partial correlation between *k*_catC_ and both *K*_C_ and *K*_C_^air^ was observed (variance explained = 21.3% and 23.3%, respectively; fig. 5*A*). Furthermore, a partial correlation was also measured between *K*_C_ and *K*_O_ (variance explained = 18.6%; fig. 5*A*). However, correlations between all other pairwise combinations of kinetic traits were found to be either marginal, or not significant (fig. 5*A*). In addition, the dependency between *K*_C_ and *K*_O_ was attenuated to 13.4% when the C_3_–C_4_, C_4_-like, and C_4_ angiosperms were excluded from the analysis (supplementary file 1, fig. S7*A*, Supplementary Material online).

Evaluation of the phylogenetic constraints revealed that they explained a significant proportion of variation in the evolution of all rubisco kinetic traits (fig. 5*B*). Moreover, the phylogenetic constraints explained a larger proportion of kinetic trait variation than catalytic trade-offs (fig. 5*C*), such that the cumulate variation explained by phylogenetic constraints (56.1%) was larger than the combined effect of all catalytic trade-offs (8.0%). Analogous results were recovered when C_3_–C_4_, C_4_-like, and C_4_ species were removed from the analysis (cumulative variance for phylogenetic constraints = 61.4%, cumulative variance for catalytic trade-offs = 6.2%; supplementary file 1, fig. S7*B* and *C*, Supplementary Material online). Thus, phylogenetic constraints have been a critical limitation on rubisco adaptation in a diverse range of photoautotrophs and have presented a greater barrier to kinetic evolution than that imposed by all catalytic trade-offs combined.

## Discussion

The evolutionary landscape of rubisco has long been proposed to be constrained by catalytic trade-offs. In support of this hypothesis, antagonistic correlations between rubisco kinetic traits inferred from studies comparing limited numbers of species are commonly cited (Tcherkez et al. 2006; Savir et al. 2010). Specifically, strong dependencies are thought to occur between rubisco specificity (*S*_C/O_), carboxylase turnover (*k*_catC_), and the Michaelis constants for CO_2_ (*K*_C_) and O_2_ (*K*_O_), respectively (Tcherkez et al. 2006; Savir et al. 2010). Combined, these trade-offs are hypothesized to limit the capacity of rubisco to assimilate CO_2_ at high rates by curtailing the inherent carboxylase activity of the enzyme, while also causing it to catalyze a reaction with O_2_ which is energetically expensive and results in a loss of fixed carbon (Bowes et al. 1971; Chollet 1977). However, all trade-offs have been inferred based on the assumption that rubisco in different species are independent (Tcherkez et al. 2006; Savir et al. 2010; Flamholz et al. 2019; Iñiguez et al. 2020). Here, we find that this assumption was incorrect and show that a significant phylogenetic signal is found in rubisco kinetic traits across the tree of life. We re-evaluated the extent of rubisco catalytic trade-offs accounting for this phylogenetic signal and found that all catalytic trade-offs were attenuated. The largest trade-offs were observed between the Michaelis constant for CO_2_ and carboxylase turnover (∼21–37%), and between the Michaelis constants for CO_2_ and O_2_ (∼9–19%), respectively. Furthermore, we demonstrated that all other catalytic trade-offs were either non-significant or substantially attenuated when the phylogenetic relationship of the species was correctly accounted for. Finally, we found that phylogenetic constraints have played a larger role than catalytic trade-offs in limiting the evolution of rubisco kinetics. Thus, rubisco kinetics have been evolving largely independently of each other in an adaptive landscape that is predominantly limited by phylogenetic constraint.

The presence of a phylogenetic signal in rubisco kinetic traits simply means that rubisco kinetics are more similar among close relatives, with this similarity changing as a function of the phylogenetic distance between species. This result is perhaps not surprising given that all extant rubisco are related by the process of descent with modification from a single ancestral enzyme (Nisbet et al. 2007). However, not all biological traits contain a phylogenetic signal (Blomberg et al. 2003; Kamilar and Cooper 2013). Furthermore, the functional consequences of changes to enzyme sequences are hard to predict (Minshull et al. 2005; Damborsky and Brezovsky 2014; Siegel et al. 2015; Carlin et al. 2016), with single amino acid substitutions often causing large effects in enzyme kinetics (Cleton-Jansen et al. 1991; Villar et al. 1997; Johnson et al. 2001). Thus, *a priori* it was unknown whether any or all of the rubisco kinetic traits would exhibit a phylogenetic signal. It will be interesting to see whether the presence of a phylogenetic signal in enzyme kinetic data is a phenomenon that is specific to rubisco, and if not it will likely be important to account for this non-independence when comparing the catalytic properties of enzymes across the tree of life.

In this work we reveal that the phylogenetic signal in rubisco kinetics is caused by phylogenetic constraint on rubisco that is independent of the catalytic trade-offs. Phylogenetic constraint in this context includes all of the processes that collectively lead to slow rates of adaptation. These processes include neutral evolution under genetic drift (Felsenstein 1985), or evolutionary stasis (Prinzing et al. 2001; Ackerly 2004; Cavender-Bares et al. 2004; Moles et al. 2005; Swenson and Enquist 2007) under which adaptive change is mitigated by processes including stabilizing selection, pleiotropy, and a lack of molecular variability or phenotypic plasticity (Maynard Smith et al. 1985; Bradshaw 1991; Edwards and Naeem 1993; Wagner 1995). Although it is possible that multiple factors contribute to the phylogenetic constraint detected in rubisco, it is likely that low molecular variability is a key driver of this phenomenon. For example, the rate of molecular evolution of rubisco is likely constrained by the requirements for 1) high levels of transcript and protein abundance (Kelly 2018; Seward and Kelly 2018), 2) maintaining complementarity to a wide array of molecular chaperones which assist in protein folding and assembly (e.g., Raf1, Raf2, RbcX, BSD2, Cpn60/Cpn20) and metabolic regulation (e.g., rubisco activase) (Carmo-Silva et al. 2015; Aigner et al. 2017), and 3) the need to preserve overall protein stability within the molecular activity-stability trade-offs (Studer et al. 2014; Duraõ et al. 2015; Cummins et al. 2018). In plants, these constraints would be further exacerbated due to the presence of the *rbcL* gene in the organellar genome that is uniparentally inherited and does not recombine (Birky 2001). For example, in angiosperms chloroplast-encoded genes evolve 10 times slower than nuclear-encoded genes (Wolfe et al. 1987; Smith 2015). Combined, these evolutionary constraints would hinder the kinetic adaptation of rubisco resulting in the phylogenetic constraint observed in this study.

The strongest catalytic trade-off detected in this study was the 21–37% dependency that was observed between *k*_catC_ and both *K*_C_ and *K*_C_^air^. This finding is compatible with the mechanistic models of rubisco (Farquhar 1979), and is supported by the recent discovery of rubisco variants which exhibit the highest *k*_catC_ ever recorded at the expense of poor CO_2_ affinities (i.e., *K*_C_ values >250 µM) (Davidi et al. 2020). Nevertheless, the dependency between CO_2_ affinity and carboxylase turnover, despite being the strongest correlation that was observed, is substantially attenuated relative to the coefficients that are conventionally cited (Tcherkez et al. 2006; Savir et al. 2010). Therefore, although selecting for a greater rubisco carboxylase turnover is evolutionarily linked with a poorer affinity for CO_2_ (higher *K*_C_), and *vice versa*, significant plasticity exists in this relationship among species such that variation in one kinetic trait only explains approximately 21–37% of variation in the other. This fact explains why there is variability in the carboxylation efficiency among angiosperm rubisco (defined as *k*_catC_/*K*_C_), a core parameter which defines the initial slope of the response of CO_2_ fixation rate to changes in CO_2_ concentration within the aerobic environment of chloroplasts in C_3_ species (Sharwood 2017). The second strongest catalytic trade-off that was observed was the 9–19% dependency between *K*_C_ and *K*_O_. This trade-off is compatible with the fact that the singular active site of rubisco binds both CO_2_ and O_2_, and thus it is plausible that mutations that affect the active site will affect biding of both substrates, though not necessarily to equivalent extents. All other catalytic trade-offs were either marginal (<9%) or non-significant. Furthermore, the combined effect of all catalytic trade-offs can only account for 6–9% of total variation in rubisco kinetics between species, a substantially smaller component than can be explained by phylogenetic constraint (30–61%).

The phylogenetically resolved analysis of rubisco kinetic evolution also identified changes in kinetic traits associated with the evolution of C_4_ photosynthesis. Specifically, *S*_C/O_ was lower in C_4_ species than in C_3_ species, whereas *k*_catC_, *K*_C_, and *K*_C_^air^ were higher in C_4_ species than in C_3_ species. Moreover, variation in *K*_O_ was found to be greater in C_4_ species than in C_3_ species. These differences in rubisco kinetics would likely be either neutral or adaptive in a C_4_ context. For example, any change in *K*_O_ would effectively be neutral under the elevated CO_2_ environment of the bundle sheath chloroplast, as it would have only a marginal effect on the *in vivo* carboxylation rate or carboxylation-to-oxygenation ratio, and thus would not cause a concomitant change to organism fitness. In contrast, an increase in *k*_catC_ in the same elevated CO_2_ environment would enable higher flux through rubisco and thus provide an energetic advantage. Accordingly, one would expect that an increased variation in *K*_O_ in C_4_ species would occur by neutral drift (Kimura 1991; Savir et al. 2010), and that an increased *k_cat_*_C_ would confer a selective advantage even if it came at the expense of a partial reduction in *K*_C_. Thus, the adaptations to rubisco kinetics that occur concomitant with the evolution of C_4_ photosynthesis are consistent with the change in CO_2_: O_2_ ratio, and the weak catalytic trade-off that exists between *k*_catC_ and *K*_C_. Here, despite the phylogenetic constraints limiting rubisco adaptation, the increased rate at which these kinetic changes occurred in C_4_ species may have been facilitated by the higher rates of molecular evolution (Kelly 2018) and diversification (Spriggs et al. 2014) that occur concomitant with the evolution of C_4_ photosynthesis.

Although every effort was taken to prevent systematic or methodological biases from influencing the results, several factors may have led to the underestimation of phylogenetic signal in the data. For example, experimental error in kinetic measurements, and/or inconsistencies associated with measurements being compiled from numerous sources, may have hindered the detection of phylogenetic signal, as has been shown in other studies (Rohlf 2001). However, to help mitigate this problem, the *S*_C/O_ values used in this analysis were normalized to avoid the discrepancy between the rates measured using an oxygen electrode assay (Parry et al. 1989) and those measured using high precision gas-phase-controlled ^3^H-RuBP-fixation assays (Kane et al. 1994) (see Materials and Methods). Thus, improvements in both the accuracy and breadth of rubisco kinetic measurements across species will lead to a concomitant improvement in our understanding of how rubisco kinetics have evolved.

Given the importance of rubisco to life on Earth, the question as to why a “perfect” rubisco has not already evolved is legitimate. For example, although rubisco *K*_C_ is thought to be near optimal in C_3_ plants in light of the 8 µM chloroplastic concentration of CO_2_ and the inherent limitations of CO_2_ as a substrate, including its inertness, hydrophobicity, and low molecular mass (Andrews and Whitney 2003; Bar-Even et al. 2011; Bathellier et al. 2018), the observed *k*_catC_ (∼3 s^−1^ per site) has often been considered low (Bar-Even et al. 2011; Tcherkez 2013; Davidi et al. 2018). In addition, all known rubisco variants catalyze a promiscuous and energetically costly reaction with O_2_. However, a recent review of rubisco kinetics relative to those of other enzymes has argued that rubisco is actually not such a bad catalyst (Bathellier et al. 2018). Indeed, the phylogenetically informed analysis of rubisco presented here demonstrates that the kinetic traits have been able to evolve largely independently of each other, with kinetic evolution primarily limited by phylogenetic constraint. These constraints induce a lag in adaptive evolution that help to explain why the enzyme is better suited to former environmental conditions.

The study presented here highlights the importance of considering phylogenetic relationships when conducting comparative analyses of enzyme kinetics across species. In doing so, it reveals that rubisco evolution has been only weakly constrained by catalytic trade-offs. Instead, phylogenetic constraints, caused by factors that limit the pace of molecular evolution, have provided a more substantial hindrance to rubisco kinetic evolution. Accordingly, it should be feasible in the current synthetic biology revolution to circumvent this evolutionary barrier on rubisco optimization. Indeed, promising steps toward this goal have been already demonstrated using directed evolution of the enzyme to generate variants with improved catalytic traits in non-photosynthetic archea (Wilson et al. 2016), photosynthetic bacteria (Zhou and Whitney 2019), and cyanobacteria (Wilson et al. 2018). Thus, our findings provide optimism for engineering rubisco in food, fiber, and fuel crops to have improved catalytic efficiency.

## Materials and Methods

### Kinetic Data

Kinetic measurements of rubisco were attained from Flamholz et al. (2019). *S*_C/O_ values measured using the O_2_ electrode method which calculate [CO_2_] using a pKa of 6.11 (Parry et al. 1989) were normalized relative to *S*_C/O_ values quantified using high precision gas-phase-controlled ^3^H-RuBP-fixation assays (Kane et al. 1994) in order to minimize methodological biases in the data. Specifically, as rubisco from wheat (*Triticum aestivum*) was measured in both the O_2_ electrode studies (Orr et al. 2016; Prins et al. 2016) as well as in the high precision method by Kane et al. (1994), multipliers were applied to all *S*_C/O_ measurements derived from O_2_ electrode assays using wheat as an enzyme standard. The distribution of *S*_C/O_ values in angiosperms before and after normalization can be seen in supplementary file 1, fig. S8, Supplementary Material online).

All kinetic traits in the data set were log transformed consistent with (Flamholz et al. 2019), and the distributional assumptions of each were verified for analyses herein. For the angiosperm focused analysis, only angiosperms with experimental measurements of all four principal kinetic traits of interest (*S*_C/O_*, k*_catC_, *K*_C_, and *K*_O_) were taken forward for subsequent analysis. However, all species in the data set with more than one kinetic trait measurement were considered for subsequent analyses of all photosynthetic organisms. In addition, an estimate of the Michaelis constant for CO_2_ under 20.95% ambient air (*K*_C_^air^) was inferred from *K*_C_ and *K*_O_ based on the formula *K*_C_ + (*K*_C_ [O_2_]/*K*_O_), where 20.95% [O_2_] in water is 253 µM.

In cases where duplicate entries for a species were present in the kinetic data set (including synonyms), the median value of their kinetic traits was used for inference. In this way, medians were also taken for *Triticum timonovum* and *Triticum timopheevii*, the former of which is a synthetic octoploid of the latter (Murashov and Morozova 2008). The modified data set containing corrected *S*_C/O_ values and no duplicate entries is provided in supplementary file 2, Supplementary Material online. Estimates of *K*_*RuBP*_are provided where available. It should also be noted that values of *k_catC_* are presented as units per active site.

### Phylogenetic Tree Inference

As sequenced genomes or transcriptomes do not exist for many species in the kinetic trait data set, whole genome phylogenomic approaches could not be used to infer the species tree necessary in order to detect a phylogenetic signal in the kinetic traits of rubisco . However, the *rbcL* gene that encodes the large subunit of rubisco has a long history of use for phylogenetic inference of species relationships (Gielly and Taberlet 1994; APG 1998, 2016) and was available for all of the angiosperms, and the majority of photosynthetic organisms, that were considered in the analyses. Accordingly, *rbcL* was selected here for use in species tree inference. The coding sequences of *rbcL* for the 137 angiosperm species for which kinetic data was available can be found in supplementary file 3, Supplementary Material online. The coding sequences for *rbcL* for the complete set of 181 photosynthetic organisms for which both kinetic data and sequencing data were available can be found in supplementary file 4, Supplementary Material online. Gene sequences were downloaded from NCBI (https://www.ncbi.nlm.nih.gov/) for all species except *Flaveria brownii* which was acquired from the 1KP database (Leebens-Mack et al. 2019). Multiple sequence alignments were performed using mafft L-INS-i (Katoh and Standley 2013), and alignments were trimmed at the terminal ends to remove unaligned positions using AliView (Larsson 2014). These trimmed nucleotide sequence alignments were used for subsequent phylogenetic analysis. Bootstrapped maximum-likelihood phylogenetic trees were inferred by IQ-TREE (Nguyen et al. 2015) using the ultrafast bootstrapping method with 1000 replicates and the Shimodaira–Hasegawa approximate-likelihood ratio branch test. The best fitting model of sequence evolution was inferred from the data automatically by IQ-TREE. The resultant maximum-likelihood phylogenetic trees were rooted manually using Dendroscope (Huson and Scornavacca 2012). A number of nodes in the angiosperm tree (supplementary file 1, fig. S1, Supplementary Material online) and a number of nodes in the tree of all photosynthetic organisms exhibited terminal zero-length branches due to 100% sequence identify with other closely related species (*n *=* *18 and *n *=* *23, respectively). These species were condensed into single data points (as their *rbcL* are 100% identical) and the mean of their kinetic traits was used. This reduced the data set to 119 angiosperms and 158 photosynthetic organisms. The phylogenetic tree inferred from the angiosperm taxa (supplementary file 1, fig. S1, Supplementary Material online) closely matched the topology of the phylogenetic tree expected from the angiosperm phylogeny with only a few alterations (APG 2016). Moreover, the topology of the phylogenetic trees inferred from the *rbcL* gene most accurately reflects the sequence similarity of rubisco, and thus were deemed as suitable for investigation of phylogenetic signal and its effects on correlations between rubisco kinetic traits.

To confirm that the phylogenetic signal was not attributable to overfitting caused by the use of the *rbcL* gene to infer the phylogeny of rubisco, an analogous maximum-likelihood phylogenetic tree was inferred using IQ-TREE (Nguyen et al. 2015) following the methods described above but based on a multiple sequence alignment in which columns containing non-synonymous nucleotide sequence changes were removed (supplementary file 5, Supplementary Material online). Due to the considerable loss of phylogenetic information accessible for tree building from this alignment, the species tree inferred using the nucleotide sequences corresponding to these ubiquitously conserved amino acid positions (supplementary file 1, fig. S2, Supplementary Material online) exhibited an additional number (*n *=* *13) of angiosperm zero-length terminal branches. As the sequences of these species are known to exhibit non-synonymous mutations which are not included in the tree, it is not appropriate to take means of their kinetic traits as above. As such, these data points were removed from the analysis using only this tree. Use of this phylogenetic tree confirmed that the presence of the phylogenetic signal in kinetic traits was not due to overfitting, however as this tree was less accurate than the full-length alignment tree, it was not used for any subsequent analysis.

### Phylogenetic Signal Analysis

The presence of a phylogenetic signal in kinetic traits was assessed using five different phylogenetic signal detection methods (Gittleman and Kot 1990; Abouheif 1999; Pagel 1999; Blomberg et al. 2003). Here, signal strength was estimated by assessing the distribution of trait values relative to the respective underlying species tree inferred from the *rbcL* sequences using methods which both depend on an explicit evolutionary model, such as Pagel’s lambda (Pagel 1999) and Blomberg’s *K* and K* (Blomberg et al. 2003), as well as the spatial autocorrelation analyses of Moran’s I (Gittleman and Kot 1990) and Abouheif’s Cmean (Abouheif 1999). Implementation of these phylogenetic signal detection tools was performed using the *phyloSignal* function of the phylosignal package (Keck et al. 2016) in the R environment. For further discussion of the differences between phylogenetic signal detection methods, see Münkemüller et al. (2012).

### Ancestral State Estimation and Mapping of Kinetic Traits to the Phylogenetic Tree

Ancestral state estimation was conducted to visualize the evolution of rubisco kinetic traits on the phylogenetic tree which relates the angiosperms. For this purpose, the kinetic data set was mapped and scaled onto the angiosperm species tree by employing the function *ggtree* in the ggtree package (Yu et al. 2017). Here, terminal branches were colored according to the measurement of the kinetic trait in the species which comprise the terminal branch, whereas internal branches were colored based on values inferred in ancestral species using ancestral state estimation (Yu et al. 2017).

### Least Squares and Linear Regression Models

All regression models between pairwise combinations of kinetic traits were fit in the R environment. Phylogenetic generalized regression accounting for the phylogenetic non-independence between species was performed using the function *pgls* in the caper package (Comparative Analyses of Phylogenetics and Evolution in R) (Orme et al. 2014). In each case, the phylogenetic signal was corrected for by using branch length transformations of the phylogenetic tree based on the mean maximum-likelihood estimates of lambda calculated for each trait, with kapa and delta held constant. In cases where the mean maximum-likelihood estimates of lambda exceeded the upper limit of the model, this value was set to 1. Phylogenetic corrections to differences in kinetic trait values between C_3_ and C_4_ plants based on the phylogenetic non-independence of species were also applied using the *pgls* function in the caper package (Orme et al. 2014) with photosynthetic type incorporated as a factorial variable.

In order to partition the variance in rubisco kinetic traits explained by phylogenetic constraints as compared with non-phylogenetic constraints, the rr2 package (Ives and Li 2018) was employed in R. Here, to assess the extent to which phylogeny can explain the variation in kinetic trait values, the explanatory power of the phylogenetic component was measured by comparing full and reduced phylogenetic regression models using the partial *R*^2^_pred_ inferential statistical, based on advice from Ives (2019). For this analysis, phylogenetic regression models were fit using the *phylolm* function in the phylolm package (Tung Ho and Ané 2014) using Pagel’s lambda model for the error term.

## Supplementary Material

Supplementary data are available at *Molecular Biology and Evolution* online.

## Supplementary Material

msab079_Supplementary_Data
