# Supplementary material for: Rubisco Adaptation Is More Limited by Phylogenetic Constraint Than by Catalytic Trade-off
Source: Mol Biol Evol. 2021 Mar 19;38(7):2880–96. doi: 10.1093/molbev/msab079 (PMC8233502; doi:10.1093/molbev/msab079)
Supplement: msab079_Supplementary_Data [file msab079_supplementary_data.zip › Supplemental_File_1.pdf]

Supplemental File 1

The phylogenetic signal in rubisco kinetic traits is not caused by overfitting due to use of rbcL for phylogenetic tree inference

Although a phylogenetic tree inferred from the nucleotide sequence of the *rbcL* gene is widely accepted as a good proxy for the true phylogenetic relationships between plant species (Gielly and Taberlet, 1994), and is used in many large scale studies of plant phylogeny (APG, 2016), there is a possibility that the use of this gene when assessing for a phylogenetic signal in rubisco kinetic traits could have resulted in overfitting. Specifically, amino acid substitutions that contribute to variation in measured rubisco kinetics would also contribute to the topology of the phylogenetic tree. Thus, to test for the presence of this potential overfitting and prevent such amino acid substitutions influencing the phylogenetic relationship between species, a phylogenetic tree was inferred for all species from a nucleotide sequence alignment of the *rbcL* gene using only ubiquitously conserved amino acid positions (i.e. sites where the amino acid was constant across all species, but the coding sequence exhibited synonymous sequence changes). The use of this phylogenetic tree containing no amino acid sequence variation (Figure S2 below) did not affect the conclusions of phylogenetic signal methods in any kinetic trait assessed. Here, an overall significant phylogenetic signal was found in all kinetic traits except K_O (Table S1 below), analogous to results previously described based on the tree inferred from the complete *rbcL* coding sequence (Table 1). Therefore, measurements of the phylogenetic signal are not an artefact of using the *rbcL* gene to infer the phylogenetic tree.

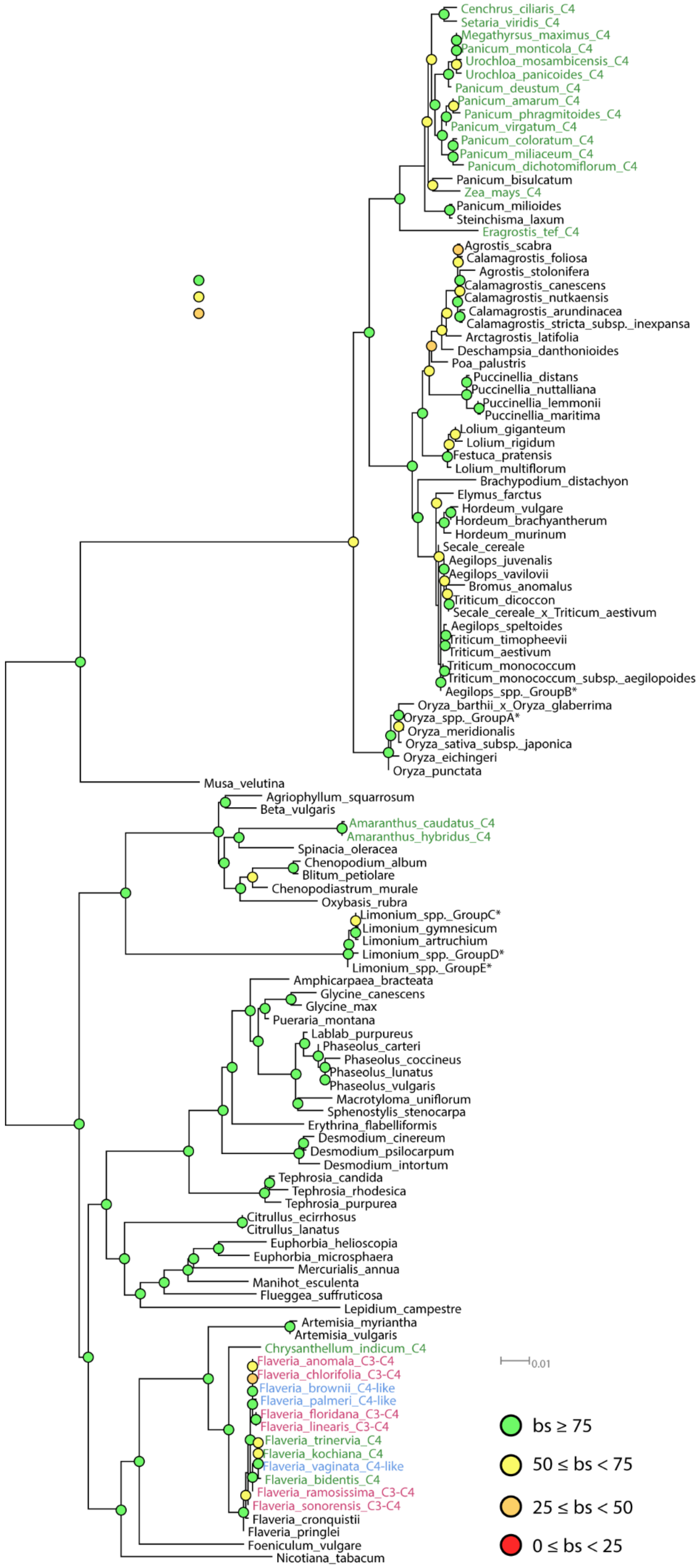
Cenchrus_ciliaris_C4
Setaria_viridis_C4
Megathyrsus_maximus_C4
Panicum_monticola_C4
Urochloa_mosambicensis_C4
Urochloa_panicoides_C4
Panicum_deustum_C4
Panicum_amarum_C4
Panicum_phragmitoides_C4
Panicum_virgatum_C4
Panicum_coloratum_C4
Panicum_miliaceum_C4
Panicum_dichotomiflorum_C4
Panicum_bisulcatum
Zea_mays_C4
Panicum_milioides
Steinchisma_laxum
Eragrostis_tef_C4
Agrostis_scabra
Calamagrostis_foliosa
Agrostis_stolonifera
Calamagrostis_canescens
Calamagrostis_nutkaensis
Calamagrostis_arundinacea
Calamagrostis_stricta_subsp._inexpansa
Arctagrostis_latifolia
Deschampsia_danthonioides
Poa_palustris
Puccinellia_distans
Puccinellia_nuttalliana
Puccinellia_lemmonii
Puccinellia_maritima
Lolium_giganteum
Lolium_rigidum
Festuca_pratensis
Lolium_multiflorum
Brachypodium_distachyon
Elymus_farctus
Hordeum_vulgare
Hordeum_brachyantherum
Hordeum_murinum
Secale_cereale
Aegilops_juvenalis
Aegilops_vavilovii
Bromus_anomalus
Triticum_dicoccon
Secale_cereale_x_Triticum_aestivum
Aegilops_speltoides
Triticum_timopheevii
Triticum_aestivum
Triticum_monococcum
Triticum_monococcum_subsp._aegilopoides
Aegilops_spp._GroupB*
Oryza_barthii_x_Oryza_glaberrima
Oryza_spp._GroupA*
Oryza_meridionalis
Oryza_sativa_subsp._japonica
Oryza_eichingeri
Oryza_punctata
Musa_velutina
Agriophyllum_squarrosum
Beta_vulgaris
Amaranthus_caudatus_C4
Amaranthus_hybridus_C4
Spinacia_oleracea
Chenopodium_album
Blitum_petiolare
Chenopodiastrum_murale
Oxybasis_rubra
Limonium_spp._GroupC*
Limonium_gymnesicum
Limonium_artruchium
Limonium_spp._GroupD*
Limonium_spp._GroupE*
Amphicarpaea_bracteata
Glycine_canescens
Glycine_max
Pueraria_montana
Lablab_purpureus
Phaseolus_carteri
Phaseolus_coccineus
Phaseolus_lunatus
Phaseolus_vulgaris
Macrotyloma_uniflorum
Sphenostylis_stenocarpa
Erythrina_flabelliformis
Desmodium_cinereum
Desmodium_psilocarpum
Desmodium_intortum
Tephrosia_candida
Tephrosia_rhodesica
Tephrosia_purpurea
Citrullus_ecirrhosus
Citrullus_lanatus
Euphorbia_helioscopia
Euphorbia_microsphaera
Mercurialis_annua
Manihot_esculenta
Flueggea_suffruticosa
Lepidium_campestre
Artemisia_myriantha
Artemisia_vulgaris
Chrysanthellum_indicum_C4
Flaveria_anomala_C3-C4
Flaveria_chlorifolia_C3-C4
Flaveria_brownii_C4-like
Flaveria_palmeri_C4-like
Flaveria_floridana_C3-C4
Flaveria_linearis_C3-C4
Flaveria_trinervia_C4
Flaveria_kochiana_C4
Flaveria_vaginata_C4-like
Flaveria_bidentis_C4
Flaveria_ramosissima_C3-C4
Flaveria_sonorensis_C3-C4
Flaveria_cronquistii
Flaveria_pringlei
Foeniculum_vulgare
Nicotiana_tabacum
0.01
bs ≥ 75
50 ≤ bs < 75
25 ≤ bs < 50
0 ≤ bs < 25

Supplementary Figure S1. Consensus phylogenetic tree of the angiosperm species in this study inferred from the nucleotide sequence of the *rbcL* gene of the large subunit of the rubisco enzyme. Bootstrap support (bs) values are indicated at internal nodes with scale indicated next to the tree. Where known, photosynthetic types other than the ancestral C_3 pathway have been annotated on to species labels which are colour coded for visualisation, and include C_3-C_4 intermediates (red), C_4-like (light green) and C_4 plants (green). Species which shared identical gene sequences have been condensed into single nodes on the tree to avoid terminal zero-length branches and are signified by asterisks. Of these, *Oryza* spp. Group A include *O. longistaminata*, *O. glaberrima*, *O. sativa* f. *spontanea*, *O. sativa* subpsp. *indica* and *O. glumipatula*; *Aegilops* spp. Group B include *A. triuncialis*, *A. uniaristata*, *A. tauschii*, *A. comosa*, *A. biuncialis*, *A. cylindrica*; *Limonium* spp. Group C include *L. antonii-llorensii*, *L. gibertii*, *L. biflorum*; *Limonium* spp. Group D include *L. echioides*, *L. barceloi*, *L. balearicum*, *L. companyonis*; and *Limonium* spp. Group E include *L. ejulabilis*, *L. retusum*, *L. leonardi-llorensii*, *L. magallufianum*, *L. grosii*.

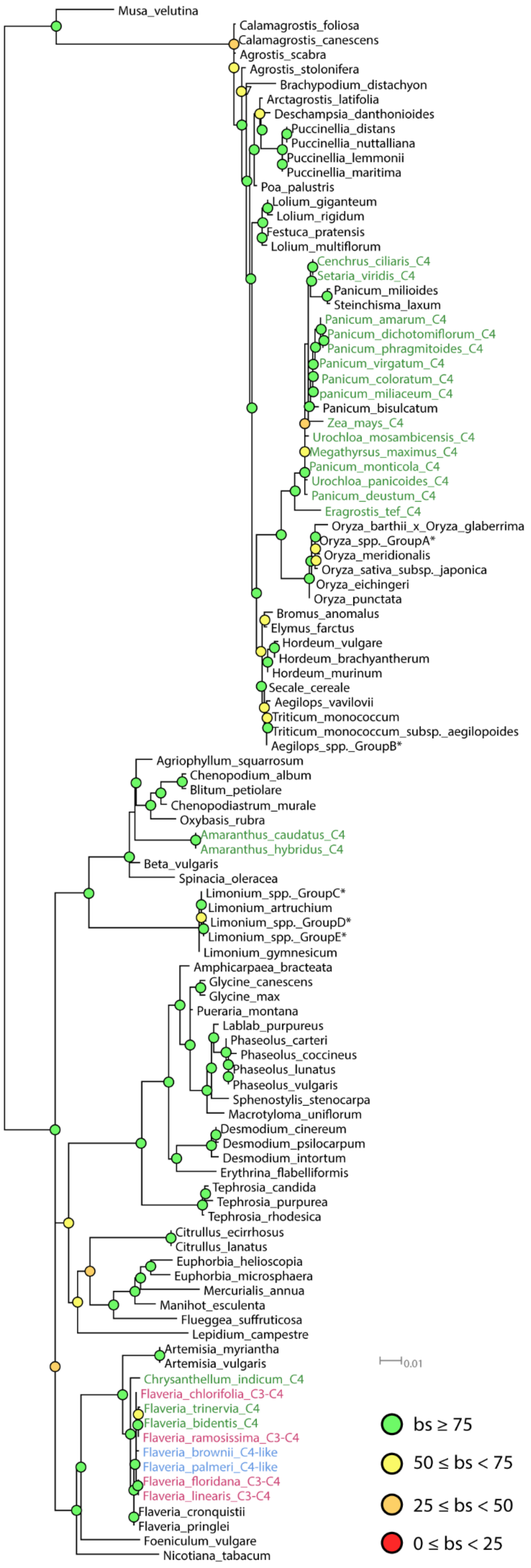
Musa_velutina
Calamagrostis_foliosa
Calamagrostis_canescens
Agrostis_scabra
Agrostis_stolonifera
Brachypodium_distachyon
Arctagrostis_latifolia
Deschampsia_danthonioides
Puccinellia_distans
Puccinellia_nuttalliana
Puccinellia_lemmonii
Puccinellia_maritima
Poa_palustris
Lolium_giganteum
Lolium_rigidum
Festuca_pratensis
Lolium_multiflorum
Cenchrus_ciliaris_C4
Setaria_viridis_C4
Panicum_milioides
Steinchisma_laxum
Panicum_amarum_C4
Panicum_dichotomiflorum_C4
Panicum_phragmitoides_C4
Panicum_virgatum_C4
Panicum_coloratum_C4
panicum_miliaceum_C4
Panicum_bisulcatum
Zea_mays_C4
Urochloa_mosambicensis_C4
Megathyrsus_maximus_C4
Panicum_monticola_C4
Urochloa_panicoides_C4
Panicum_deustum_C4
Eragrostis_tef_C4
Oryza_barthii_x_Oryza_glaberrima
Oryza_spp._GroupA*
Oryza_meridionalis
Oryza_sativa_subsp._japonica
Oryza_eichingeri
Oryza_punctata
Bromus_anomalus
Elymus_farctus
Hordeum_vulgare
Hordeum_brachyantherum
Hordeum_murinum
Secale_cereale
Aegilops_vavilovii
Triticum_monococcum
Triticum_monococcum_subsp._aegilopoides
Aegilops_spp._GroupB*
Agriophyllum_squarrosum
Chenopodium_album
Blitum_petiolare
Chenopodiastrum_murale
Oxybasis_rubra
Amaranthus_caudatus_C4
Amaranthus_hybridus_C4
Beta_vulgaris
Spinacia_oleracea
Limonium_spp._GroupC*
Limonium_artruchium
Limonium_spp._GroupD*
Limonium_spp._GroupE*
Limonium_gymnesicum
Amphicarpaea_bracteata
Glycine_canescens
Glycine_max
Pueraria_montana
Lablab_purpureus
Phaseolus_carteri
Phaseolus_coccineus
Phaseolus_lunatus
Phaseolus_vulgaris
Sphenostylis_stenocarpa
Macrotyloma_uniflorum
Desmodium_cinereum
Desmodium_psilocarpum
Desmodium_intortum
Erythrina_flabelliformis
Tephrosia_candida
Tephrosia_purpurea
Tephrosia_rhodesica
Citrullus_ecirrhosus
Citrullus_lanatus
Euphorbia_helioscopia
Euphorbia_microsphaera
Mercurialis_annua
Manihot_esculenta
Flueggea_suffruticosa
Lepidium_campestre
Artemisia_myriantha
Artemisia_vulgaris
Chrysanthellum_indicum_C4
Flaveria_chlorifolia_C3-C4
Flaveria_trinervia_C4
Flaveria_bidentis_C4
Flaveria_ramosissima_C3-C4
Flaveria_brownii_C4-like
Flaveria_palmeri_C4-like
Flaveria_floridana_C3-C4
Flaveria_linearis_C3-C4
Flaveria_cronquistii
Flaveria_pringlei
Foeniculum_vulgare
Nicotiana_tabacum
0.01
bs ≥ 75
50 ≤ bs < 75
25 ≤ bs < 50
0 ≤ bs < 25

Supplementary Figure S2. Consensus phylogenetic tree of the angiosperm species in this study inferred from the nucleotide sequence of the *rbcL* gene of the large subunit of the rubisco enzyme when including only sites which encode ubiquitously conserved amino acid positions. The confidence of branch points on the tree are depicted as nodes which are colour coded by bootstrap support (bs) values. Where known, photosynthetic types other than the ancestral C_3 pathway have been annotated on to species labels which are colour coded for visualisation, and include C_3-C_4 intermediates (red), C_4-like (blue) and C_4 plants (green). Species which shared identical sequences when considering the entire sequence length have been condensed into single nodes on the tree to avoid terminal zero-length branches and are signified by asterisks (see supplementary Figure S1 for further details). In addition to these, a number of terminal zero length branches were present between nodes on the tree as an artefact of removing non-synonymous positions in the *rbcL* sequence alignment. All but one of the species within these respective groups of plants containing zero length branches were removed and are thus not present in this tree, including *Aegilops juvenalis*, *Aegilops speltoides*, *Calamagrostis arundinacea*, *Calamagrostis stricta subsp. inexpansa*, *Calamagrostis nutkaensis*, *Flaveria anomala*, *Flaveria kochiana*, *Flaveria sonorensis*, *Flaveria vaginata*, *Triticum dicoccon*, *Triticum timopheevii*, *Secale cereale x Triticum aestivum* and *Triticum aestivum*

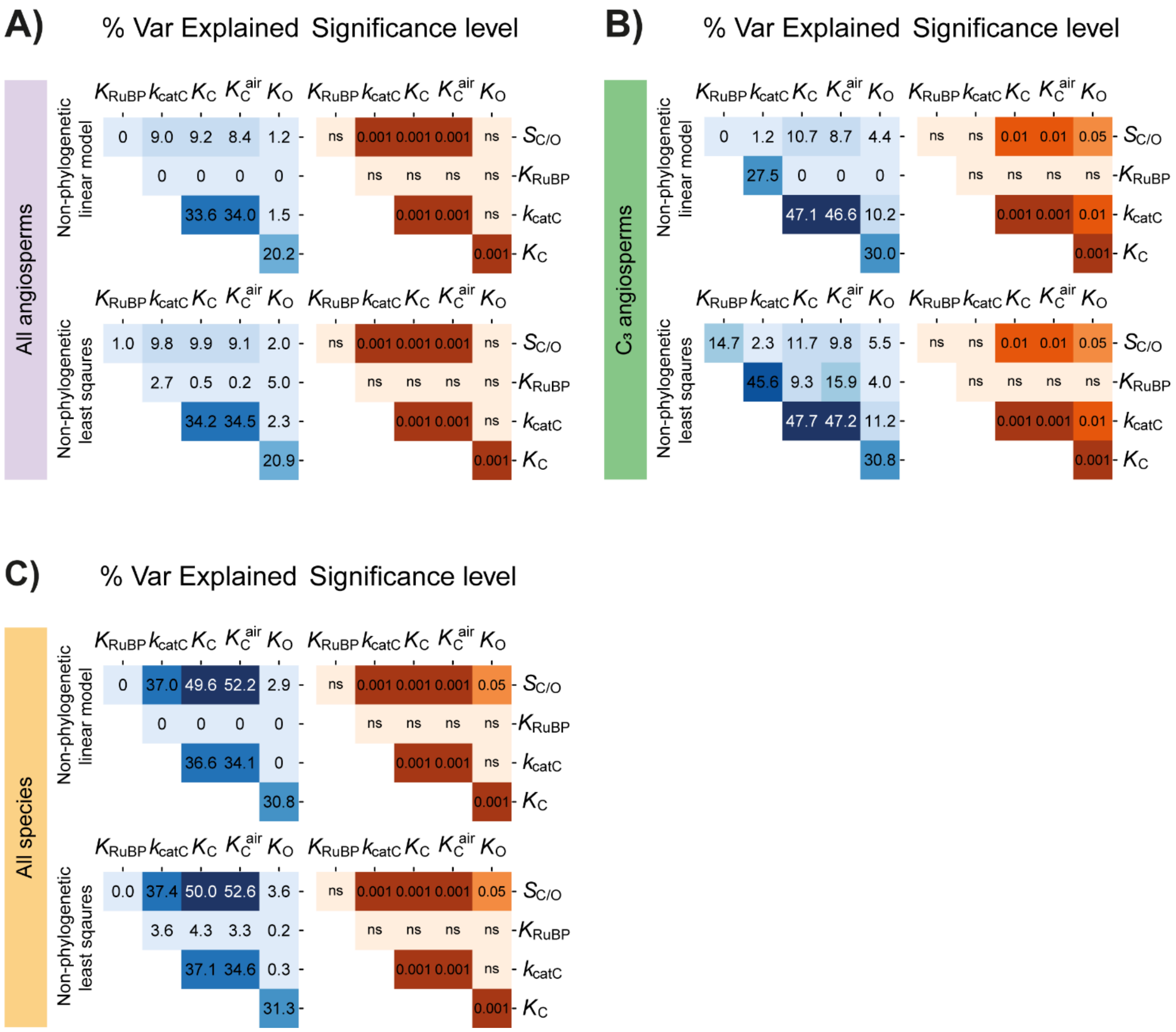
A)
% Var Explained
Significance level
All angiosperms
Non-phylogenetic linear model
Non-phylogenetic least sqaures
B)
% Var Explained
Significance level
C3 angiosperms
Non-phylogenetic linear model
Non-phylogenetic least sqaures
C)
% Var Explained
Significance level
All species
Non-phylogenetic linear model
Non-phylogenetic least sqaures

Supplementary Figure S3. A) Pairwise correlation coefficients (percent variance explained) and associated p-values between different rubisco kinetic traits assessed across the complete set of angiosperms using either non-phylogenetic linear models or least squares regression models. Significance values are represented as α levels, where; $\alpha = 0.001$ if $p < 0.001$, $\alpha = 0.01$ if $0.001 < p < 0.01$, $\alpha = 0.05$ if $0.01 < p < 0.05$, and α = ns if $p > 0.05$. **B)** as in A but for C_3 angiosperms only. **C)** as in A and B but for the complete set of photosynthetic organisms.

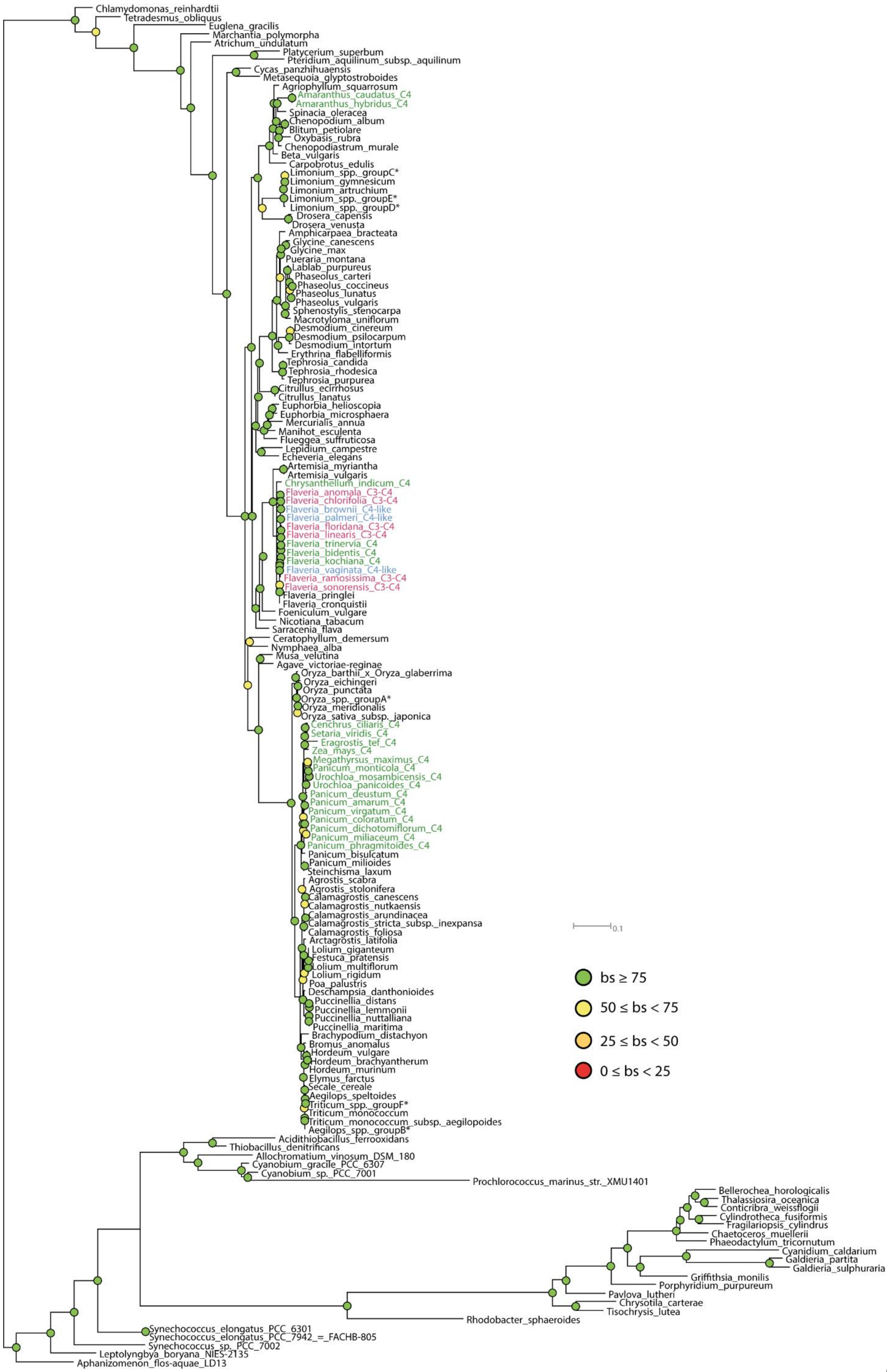

Chlamydomonas_reinhardtii
Tetradesmus_obliquus
Euglena_gracilis
Marchantia_polymorpha
Atrichum_undulatum
Platycerium_superbum
Pteridium_aquilinum_subsp._aquilinum
Cycas_panzhihuaensis
Metasequoia_glyptostroboides
Agriophyllum_squarrosum
Amaranthus_caudatus_C4
Amaranthus_hybridus_C4
Spinacia_oleracea
Chenopodium_album
Blitum_petiolare
Oxybasis_rubra
Chenopodiastrum_murale
Beta_vulgaris
Carpobrotus_edulis
Limonium_spp._groupC*
Limonium_gymnesicum
Limonium_artruchium
Limonium_spp._groupE*
Limonium_spp._groupD*
Drosera_capensis
Drosera_venusta
Amphicarpaea_bracteata
Glycine_canescens
Glycine_max
Pueraria_montana
Lablab_purpureus
Phaseolus_carteri
Phaseolus_coccineus
Phaseolus_lunatus
Phaseolus_vulgaris
Sphenostylis_stenocarpa
Macrotyloma_uniflorum
Desmodium_cinereum
Desmodium_psilocarpum
Desmodium_intortum
Erythrina_flabelliformis
Tephrosia_candida
Tephrosia_rhodesica
Tephrosia_purpurea
Citrullus_ecirrhosus
Citrullus_lanatus
Euphorbia_helioscopia
Euphorbia_microsphaera
Mercurialis_annua
Manihot_esculenta
Flueggea_suffruticosa
Lepidium_campestre
Echeveria_elegans
Artemisia_myriantha
Artemisia_vulgaris
Chrysanthellum_indicum_C4
Flaveria_anomala_C3-C4
Flaveria_chlorifolia_C3-C4
Flaveria_brownii_C4-like
Flaveria_palmeri_C4-like
Flaveria_floridana_C3-C4
Flaveria_linearis_C3-C4
Flaveria_trinervia_C4
Flaveria_bidentis_C4
Flaveria_kochiana_C4
Flaveria_vaginata_C4-like
Flaveria_ramosissima_C3-C4
Flaveria_sonorensis_C3-C4
Flaveria_pringlei
Flaveria_cronquistii
Foeniculum_vulgare
Nicotiana_tabacum
Sarracenia_flava
Ceratophyllum_demersum
Nymphaea_alba
Musa_velutina
Agave_victoriae-reginae
Oryza_barthii_x_Oryza_glaberrima
Oryza_eichingeri
Oryza_punctata
Oryza_spp._groupA*
Oryza_meridionalis
Oryza_sativa_subsp._japonica
Cenchrus_ciliaris_C4
Setaria_viridis_C4
Eragrostis_tef_C4
Zea_mays_C4
Megathyrsus_maximus_C4
Panicum_monticola_C4
Urochloa_mosambicensis_C4
Urochloa_panicoides_C4
Panicum_deustum_C4
Panicum_amarum_C4
Panicum_virgatum_C4
Panicum_coloratum_C4
Panicum_dichotomiflorum_C4
Panicum_miliaceum_C4
Panicum_phragmitoides_C4
Panicum_bisulcatum
Panicum_milioides
Steinchisma_laxum
Agrostis_scabra
Agrostis_stolonifera
Calamagrostis_canescens
Calamagrostis_nutkaensis
Calamagrostis_arundinacea
Calamagrostis_stricta_subsp._inexpansa
Calamagrostis_foliosa
Arctagrostis_latifolia
Lolium_giganteum
Festuca_pratensis
Lolium_multiflorum
Lolium_rigidum
Poa_palustris
Deschampsia_danthonioides
Puccinellia_distans
Puccinellia_lemmonii
Puccinellia_nuttalliana
Puccinellia_maritima
Brachypodium_distachyon
Bromus_anomalus
Hordeum_vulgare
Hordeum_brachyantherum
Hordeum_murinum
Elymus_farctus
Secale_cereale
Aegilops_speltoides
Triticum_spp._groupF*
Triticum_monococcum
Triticum_monococcum_subsp._aegilopoides
Aegilops_spp._groupB*
Acidithiobacillus_ferrooxidans
Thiobacillus_denitrificans
Allochromatium_vinosum_DSM_180
Cyanobium_gracile_PCC_6307
Cyanobium_sp._PCC_7001
Prochlorococcus_marinus_str._XMU1401
Bellerochea_horologicalis
Thalassiosira_oceanica
Conticribra_weissflogii
Cylindrotheca_fusiformis
Fragilariopsis_cylindrus
Chaetoceros_muellerii
Phaeodactylum_tricornutum
Cyanidium_caldarium
Galdieria_partita
Galdieria_sulphuraria
Griffithsia_monilis
Porphyridium_purpureum
Pavlova_lutheri
Chrysotila_carterae
Tisochrysis_lutea
Rhodobacter_sphaeroides
Synechococcus_elongatus_PCC_6301
Synechococcus_elongatus_PCC_7942_=_FACHB-805
Synechococcus_sp._PCC_7002
Leptolyngbya_boryana_NIES-2135
Aphanizomenon_flos-aquae_LD13
0.1
bs ≥ 75
50 ≤ bs < 75
25 ≤ bs < 50
0 ≤ bs < 25

67

Supplementary Figure S4. Consensus phylogenetic tree of all photosynthetic organisms in this study inferred from the nucleotide sequence of the *rbcL* gene of the large subunit of the rubisco enzyme. The confidence of branch points on the tree are depicted as nodes which are colour coded by bootstrap support (bs) values. Where known, photosynthetic types other than the ancestral C_3 pathway have been annotated on to species labels which are colour coded for visualisation, and include C_3-C_4 intermediates (red), C_4-like (blue) and C_4 plants (green). Species which shared identical sequences when considering the entire sequence length have been condensed into single nodes on the tree to avoid terminal zero-length branches and are signified by asterisks (see supplementary Figure S1 for further details). In addition to those species mentioned in supplementary Figure S1, an additional number of species exhibited terminal zero length branches relative to other nodes in this tree due to more stringent trimming of non-aligned positions at the terminal ends of the sequence alignment of all photosynthetic organisms. Of these additional species which exhibited terminal zero length branches, *Aegilops juvenalis* and *Aegilops vavilovii* were consensed into the node *Aegilops* spp. Group B, and *Triticum dicoccon, Secale cereale x Triticum aestivum*, *Triticum aestivum* and *Triticum timopheevii* were condensed into the node *Triticum* spp. Group F.

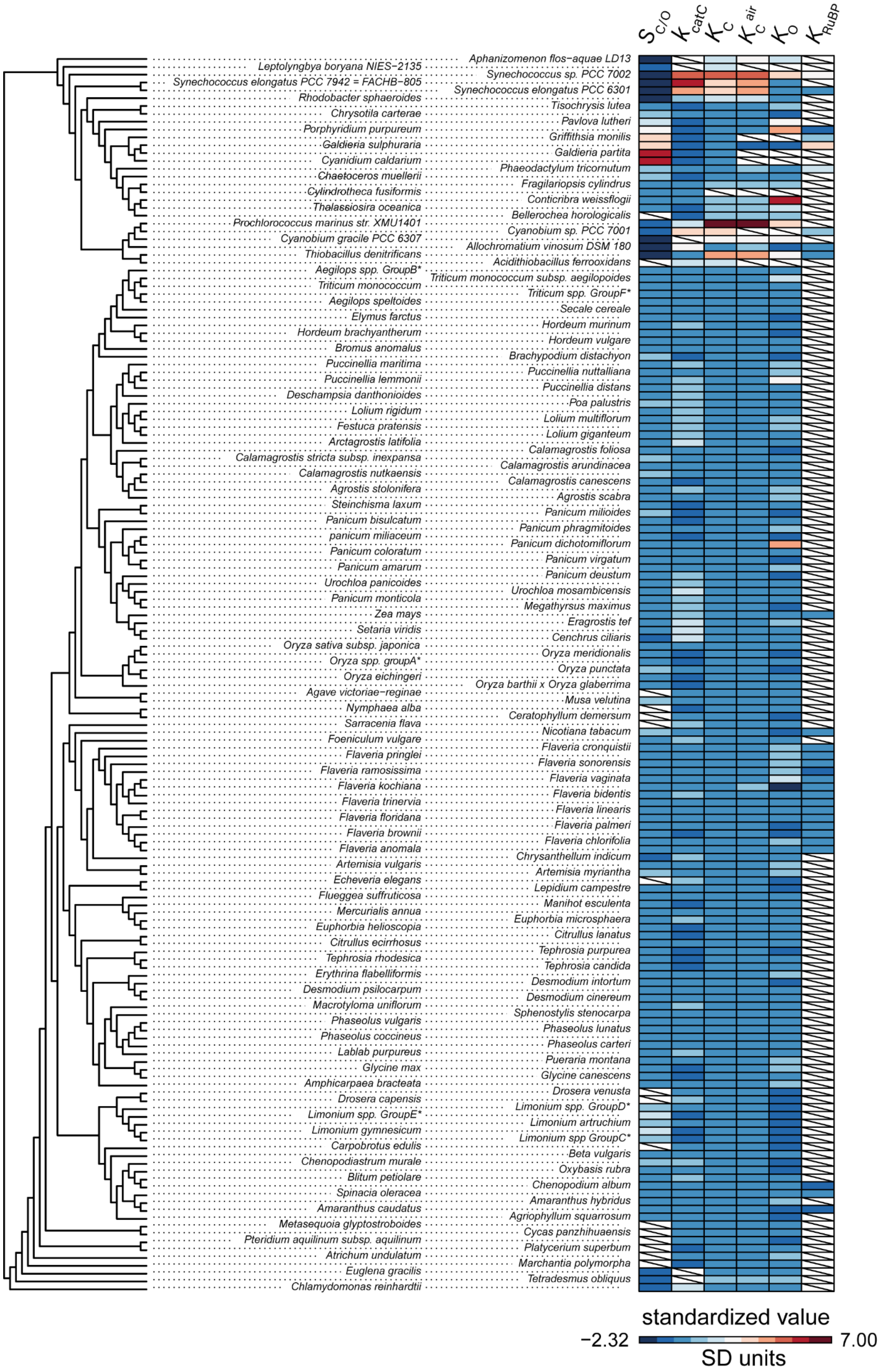
$S_{C/O}$
K_{catC}
K_C
K_C^{air}
K_O
K_{RuBP}
Aphanizomenon flos−aquae LD13
Leptolyngbya boryana NIES−2135
Synechococcus sp. PCC 7002
Synechococcus elongatus PCC 7942 = FACHB−805
Synechococcus elongatus PCC 6301
Rhodobacter sphaeroides
Tisochrysis lutea
Chrysotila carterae
Pavlova lutheri
Porphyridium purpureum
Griffithsia monilis
Galdieria sulphuraria
Galdieria partita
Cyanidium caldarium
Phaeodactylum tricornutum
Chaetoceros muellerii
Fragilariopsis cylindrus
Cylindrotheca fusiformis
Conticribra weissflogii
Thalassiosira oceanica
Bellerochea horologicalis
Prochlorococcus marinus str. XMU1401
Cyanobium sp. PCC 7001
Cyanobium gracile PCC 6307
Allochromatium vinosum DSM 180
Thiobacillus denitrificans
Acidithiobacillus ferrooxidans
Aegilops spp. GroupB*
Triticum monococcum subsp. aegilopoides
Triticum monococcum
Triticum spp. GroupF*
Aegilops speltoides
Secale cereale
Elymus farctus
Hordeum murinum
Hordeum brachyantherum
Hordeum vulgare
Bromus anomalus
Brachypodium distachyon
Puccinellia maritima
Puccinellia nuttalliana
Puccinellia lemmonii
Puccinellia distans
Deschampsia danthonioides
Poa palustris
Lolium rigidum
Lolium multiflorum
Festuca pratensis
Lolium giganteum
Arctagrostis latifolia
Calamagrostis foliosa
Calamagrostis stricta subsp. inexpansa
Calamagrostis arundinacea
Calamagrostis nutkaensis
Calamagrostis canescens
Agrostis stolonifera
Agrostis scabra
Steinchisma laxum
Panicum milioides
Panicum bisulcatum
Panicum phragmitoides
panicum miliaceum
Panicum dichotomiflorum
Panicum coloratum
Panicum virgatum
Panicum amarum
Panicum deustum
Urochloa panicoides
Urochloa mosambicensis
Panicum monticola
Megathyrsus maximus
Zea mays
Eragrostis tef
Setaria viridis
Cenchrus ciliaris
Oryza sativa subsp. japonica
Oryza meridionalis
Oryza spp. groupA*
Oryza punctata
Oryza eichingeri
Oryza barthii x Oryza glaberrima
Agave victoriae−reginae
Musa velutina
Nymphaea alba
Ceratophyllum demersum
Sarracenia flava
Nicotiana tabacum
Foeniculum vulgare
Flaveria cronquistii
Flaveria pringlei
Flaveria sonorensis
Flaveria ramosissima
Flaveria vaginata
Flaveria kochiana
Flaveria bidentis
Flaveria trinervia
Flaveria linearis
Flaveria floridana
Flaveria palmeri
Flaveria brownii
Flaveria chlorifolia
Flaveria anomala
Chrysanthellum indicum
Artemisia vulgaris
Artemisia myriantha
Echeveria elegans
Lepidium campestre
Flueggea suffruticosa
Manihot esculenta
Mercurialis annua
Euphorbia microsphaera
Euphorbia helioscopia
Citrullus lanatus
Citrullus ecirrhosus
Tephrosia purpurea
Tephrosia rhodesica
Tephrosia candida
Erythrina flabelliformis
Desmodium intortum
Desmodium psilocarpum
Desmodium cinereum
Macrotyloma uniflorum
Sphenostylis stenocarpa
Phaseolus vulgaris
Phaseolus lunatus
Phaseolus coccineus
Phaseolus carteri
Lablab purpureus
Pueraria montana
Glycine max
Glycine canescens
Amphicarpaea bracteata
Drosera venusta
Drosera capensis
Limonium spp. GroupD*
Limonium spp. GroupE*
Limonium artruchium
Limonium gymnesicum
Limonium spp GroupC*
Carpobrotus edulis
Beta vulgaris
Chenopodiastrum murale
Oxybasis rubra
Chenopodium album
Blitum petiolare
Spinacia oleracea
Amaranthus hybridus
Amaranthus caudatus
Agriophyllum squarrosum
Metasequoia glyptostroboides
Cycas panzhihuaensis
Pteridium aquilinum subsp. aquilinum
Platycerium superbum
Atrichum undulatum
Marchantia polymorpha
Euglena gracilis
Tetradesmus obliquus
Chlamydomonas reinhardtii
standardized value
−2.32
7.00
SD units

85

Supplementary Figure S5. Heatmap depicting the normalised variation in rubisco kinetic traits across all studied photosynthetic organisms (± S.D. away from each respective kinetic trait mean).

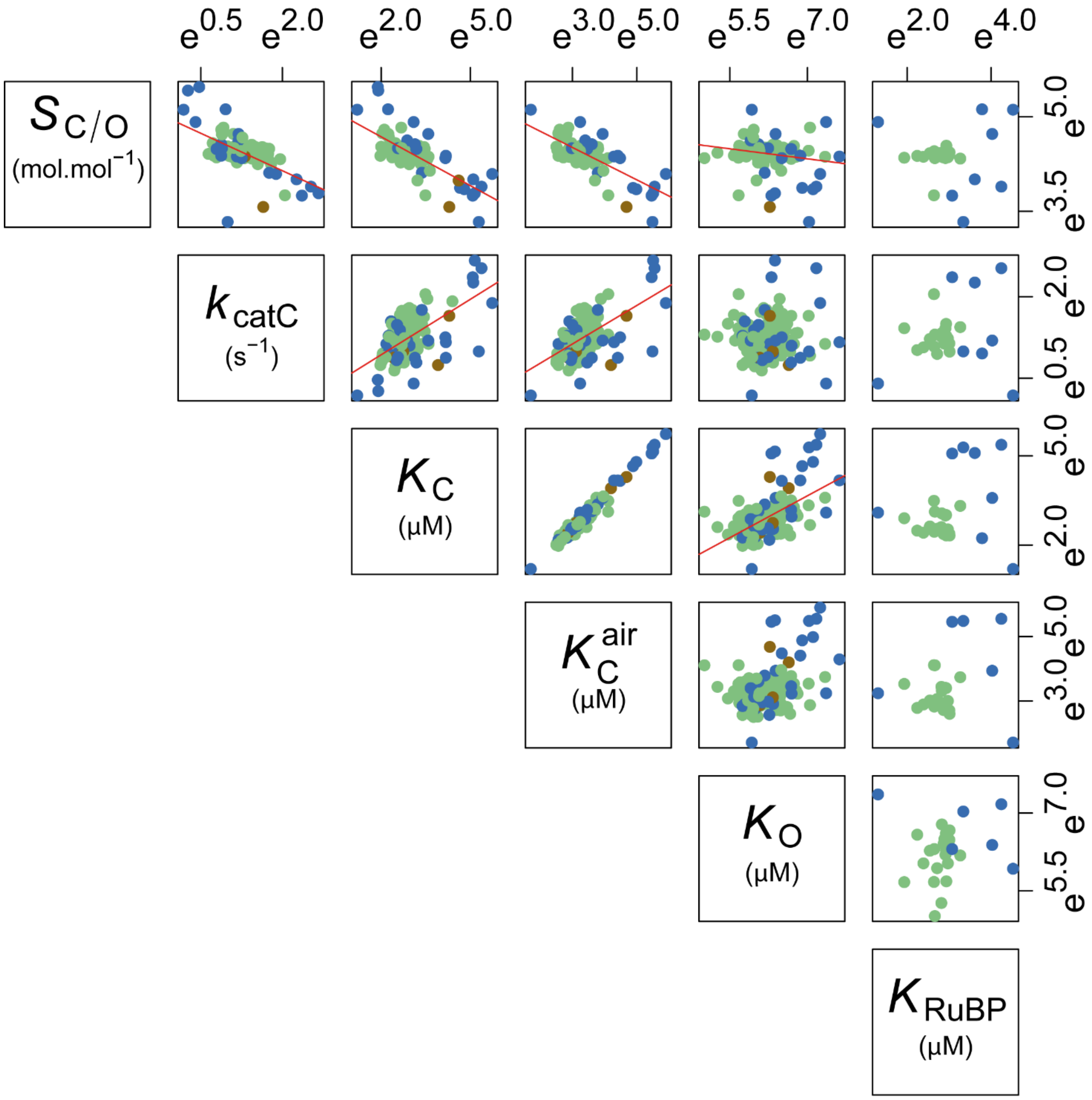

$S_{C/O}$ (mol.mol^{-1})
k_{catC} (s^{-1})
K_C (µM)
K_C^{air} (µM)
K_O (µM)
K_{RuBP} (µM)
$e^{0.5}$ $e^{2.0}$
$e^{2.0}$ $e^{5.0}$
$e^{3.0}$ $e^{5.0}$
$e^{5.5}$ $e^{7.0}$
$e^{2.0}$ $e^{4.0}$
$e^{3.5}$ $e^{5.0}$
$e^{0.5}$ $e^{2.0}$
$e^{2.0}$ $e^{5.0}$
$e^{3.0}$ $e^{5.0}$
$e^{5.5}$ $e^{7.0}$

89

Supplementary Figure S6. The trends in relationships between all pairwise combinations of log transformed rubisco kinetic traits across all studied photosynthetic organisms. Data points are colour coded by taxonomic group (plant angiosperm rubisco: green, plant non-angiosperm rubisco: brown, and non-plant rubisco: blue).

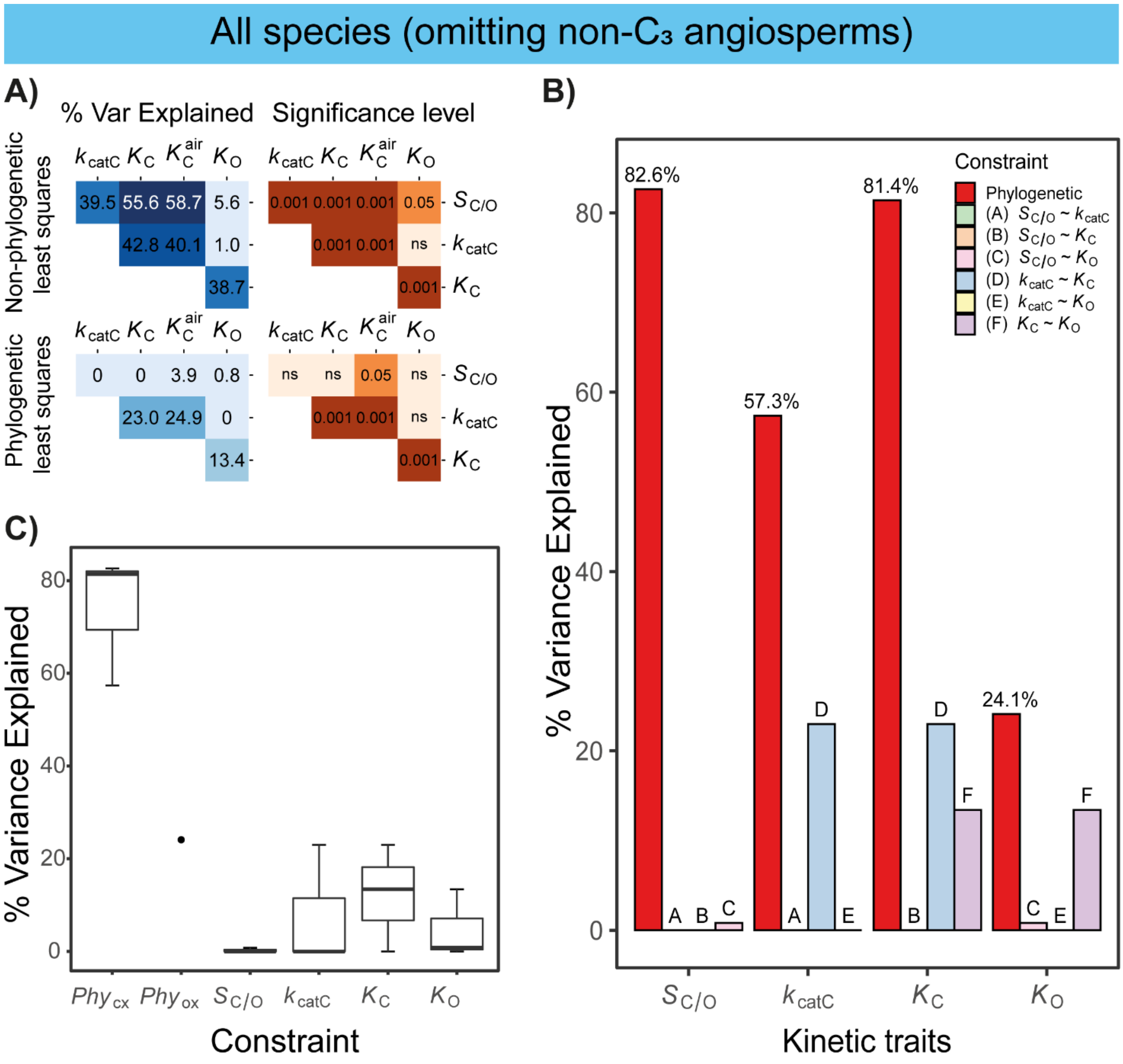

All species (omitting non-C3 angiosperms)
A)
% Var Explained
Significance level
Non-phylogenetic least squares
Phylogenetic least squares
kcatC KC KCair KO
SC/O
kcatC
KC
39.5 55.6 58.7 5.6
42.8 40.1 1.0
38.7
0.001 0.001 0.001 0.05
0.001 0.001 ns
0.001
0 0 3.9 0.8
23.0 24.9 0
13.4
ns ns 0.05 ns
0.001 0.001 ns
0.001
B)
Constraint
Phylogenetic
(A) SC/O ~ kcatC
(B) SC/O ~ KC
(C) SC/O ~ KO
(D) kcatC ~ KC
(E) kcatC ~ KO
(F) KC ~ KO
82.6%
57.3%
81.4%
24.1%
% Variance Explained
Kinetic traits
C)
% Variance Explained
Phycx Phyox SC/O kcatC KC KO
Constraint

Supplementary Figure S7. Kinetic and phylogenetic constraints on rubisco adaptation across all studied photosynthetic organisms omitting known C_3-C_4, C_4-like and C_4 angiosperms **A)** Pairwise correlation coefficients (percent variance explained) and associated *p*-values between different rubisco kinetic traits assessed using non-phylogenetic least squares regression models or phylogenetic least squares regression models. Significance values are represented as α levels, where; α = 0.001 if $p < 0.001$, α = 0.01 if $0.001 < p < 0.01$, α = 0.05 if $0.01 < p < 0.05$, and α = ns if $p > 0.05$. **B)** The variation (%) in rubisco kinetic traits across photosynthetic organisms (omitting non-C_3 angiosperms) that can be explained by phylogenetic constraint and each catalytic trade-off. **C)** Boxplot of all variation explained in each kinetic trait by kinetic trait correlations in comparison to variation explained by phylogeny in all photosynthetic organisms. The phylogenetic constraints on the carboxylase-related traits Phy_{CX} (includes $Phy_{S_{c/o}}$, $Phy_{K_{catc}}$, and Phy_{K_c}) and phylogenetic constraints on the oxygenase-related trait Phy_{ox} (includes Phy_{K_o} only) are presented separately.

112

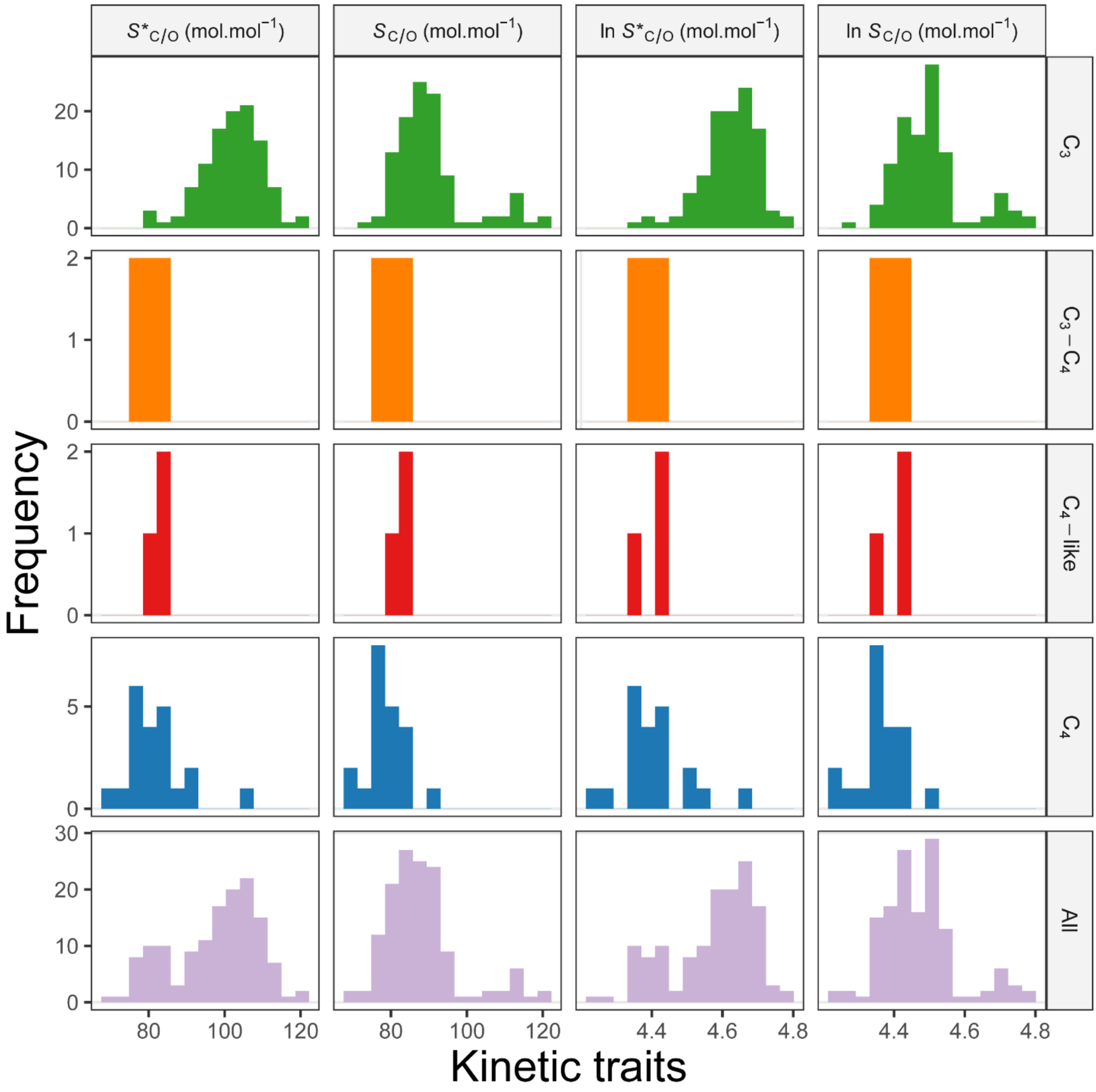
S*c/o (mol.mol−1)
Sc/o (mol.mol−1)
ln S*c/o (mol.mol−1)
ln Sc/o (mol.mol−1)
C3
C3 − C4
C4 − like
C4
All
Frequency
Kinetic traits

113

Supplementary Figure S8. The distributions of values for rubisco specificity in angiosperms before ($S^*_{C/O}$) and after ($S_{C/O}$) normalisation of measurements using the O_2 electrode method (Orr *et al.*, 2016; Prins *et al.*, 2016) relative to those quantified using high precision gas-phase-controlled ^{3}H-RuBP-fixation assays (Kane *et al.*, 1994). Species are grouped by their photosynthetic types (rows). Plants have been classified as those which perform C_3 photosynthesis (C_3; *n* = 107), C_4 photosynthesis (C_4; *n* = 6), C_3-C_4 intermediate (C_3-C_4; *n* = 3), C_4-like (C_4-like; *n* = 21). Both non-transformed and log-transformed non-normalised and normalised specificity values are shown, where respective units are shown in column labels.

Supplementary Table S1. The phylogenetic signal strength and associated significance level in rubisco kinetic traits across the phylogenetic tree in Supplementary Figure S2 in five signal detection methods. Statistics are rounded to 3 decimal places, and significance values are represented as α levels, where; α = 0.001 if $p < 0.001$, α = 0.01 if $0.001 < p < 0.01$, α = 0.05 if $0.01 < p < 0.05$, and α = ns if $p > 0.05$.

**Kinetic Trait**	**C mean**		**I**		**K**		**K ***		**Lambda**	
	Stat	***α***	***Stat***	***α***	***Stat***	***α***	***Stat***	***α***	***Stat***	***α***
$S_{C/O}$	0.502	0.001	0.516	0.001	0.001	0.05	0.001	0.05	0.873	0.001
k_{CatC}	0.343	0.001	0.303	0.001	0.002	0.001	0.003	0.001	0.940	0.05
K_C	0.344	0.001	0.339	0.001	0.002	0.01	0.002	0.001	0.948	0.001
K_C^{air}	0.295	0.001	0.291	0.001	0.002	0.001	0.002	0.001	0.52	0.05
K_O	0.125	0.05	0.142	0.05	0	ns	0	ns	0	ns

Supplementary Table S2. Significance level of assessed differences in rubisco kinetic traits between C_3 and C_4 angiosperms compared when measured in the absence of the phylogenetic tree, and when correctly accounting for the phylogenetic non-independence of species. Significance values are represented as α levels, where; α = 0.001 if $p < 0.001$, α = 0.01 if $0.001 < p < 0.01$, α = 0.05 if $0.01 < p < 0.05$, and α = ns if $p > 0.05$

**Kinetic Trait**	***α***	
	**– Phylogenetic information**	**+ Phylogenetic information**
$S_{C/O}$	0.001	0.001
k_{CatC}	0.001	0.001
K_C	0.05	0.001
K_C^{air}	0.01	0.001
K_O	ns	ns

Supplementary Table S3. The phylogenetic signal strength and associated significance level in rubisco kinetic traits across all studied photosynthetic organisms omitting known C_3-C_4, C_4-like and C_4 angiosperms using five different signal detection methods. Statistics are rounded to 3 decimal places and significance values are represented as α levels, where; α = 0.001 if $p < 0.001$, α = 0.01 if $0.001 < p < 0.01$, α = 0.05 if $0.01 < p < 0.05$, and α = ns if $p > 0.05$.

**Kinetic Trait**	**C mean**		**I**		**K**		**K ***		**Lambda**	
	Stat	***α***	***Stat***	***α***	***Stat***	***α***	***Stat***	***α***	***Stat***	***α***
$S_{C/O}$	0.717	0.001	0.245	0.001	0.006	0.001	0.003	0.001	1.009	0.001
k_{CatC}	0.53	0.001	0.313	0.001	0.002	0.001	0.001	0.01	0.955	0.001
K_C	0.75	0.001	0.309	0.001	0.005	0.001	0.002	0.001	0.986	0.001
K_C^{air}	0.657	0.001	0.327	0.001	0.005	0.001	0.002	0.001	0.983	0.001
K_O	0.332	0.001	0.151	0.01	0	0.05	0	ns	0.932	0.001

Supplementary Table S4. Key of abbreviated species labels as used in Figure 2 and Figure 3A.

Code	Species
A.juv	*Aegilops juvenalis*
A.spe	*Aegilops speltoides*
A.vav	*Aegilops vavilovii*
A.squ	*Agriophyllum squarrosum*
A.sca	*Agrostis scabra*
A.sto	*Agrostis stolonifera*
A.cau	*Amaranthus caudatus*
A.hyb	*Amaranthus hybridus*
A.bra	*Amphicarpaea bracteata*
A.lat	*Arctagrostis latifolia*
A.myr	*Artemisia myriantha*
A.vul	*Artemisia vulgaris*
B.dis	*Brachypodium distachyon*
B.vul	*Beta vulgaris*
B.ano	*Bromus anomalus*
C.aru	*Calamagrostis arundinacea*
C.can	*Calamagrostis canescens*
C.fol	*Calamagrostis foliosa*
C.str	*Calamagrostis stricta subsp. inexpansa*
C.nut	*Calamagrostis nutkaensis*
C.cil	*Cenchrus ciliaris*
C.alb	*Chenopodium album*
C.mur	*Chenopodiastrum murale*
B.pet	*Blitum petiolare*
O.rub	*Oxybasis rubra*
C.ind	*Chrysanthellum indicum*
C.eci	*Citrullus ecirrhosus*
C.lan	*Citrullus lanatus*
D.dan	*Deschampsia danthonioides*
D.cin	*Desmodium cinereum*
D.int	*Desmodium intortum*
D.psi	*Desmodium psilocarpum*
E.far	*Elymus farctus*
E.tef	*Eragrostis tef*
E.fla	*Erythrina flabelliformis*
E.hel	*Euphorbia helioscopia*
E.mic	*Euphorbia microsphaera*
L.gig	*Lolium giganteum*
F.pra	*Festuca pratensis*
F.ano	*Flaveria anomala*
F.tri	*Flaveria trinervia*
F.bid	*Flaveria bidentis*
F.bro	*Flaveria brownii*
F.chl	*Flaveria chlorifolia*
F.cro	*Flaveria cronquistii*

F.flo	*Flaveria floridana*
F.koc	*Flaveria kochiana*
F.lin	*Flaveria linearis*
F.pal	*Flaveria palmeri*
F.pri	*Flaveria pringlei*
F.ram	*Flaveria ramosissima*
F.son	*Flaveria sonorensis*
F.vag	*Flaveria vaginata*
F.suf	*Flueggea suffruticosa*
F.vul	*Foeniculum vulgare*
G.can	*Glycine canescens*
G.max	*Glycine max*
H.vul	*Hordeum vulgare*
H.bra	*Hordeum brachyantherum*
H.mur	*Hordeum murinum*
L.pur	*Lablab purpureus*
L.cam	*Lepidium campestre*
L.artr	*Limonium artruchium*
L.gym	*Limonium gymnesicum*
L.mul	*Lolium multiflorum*
L.rig	*Lolium rigidum*
M.uni	*Macrotyloma uniflorum*
M.esc	*Manihot esculenta*
M.max	*Megathyrsus maximus*
M.ann	*Mercurialis annua*
M.vel	*Musa velutina*
N.tab	*Nicotiana tabacum*
xO.hyb	*Oryza barthii x Oryza glaberrima*
O.eic	*Oryza eichingeri*
O.mer	*Oryza meridionalis*
O.pun	*Oryza punctata*
O.sat	*Oryza sativa* subsp. *japonica*
P.ama	*Panicum amarum*
P.bis	*Panicum bisulcatum*
P.col	*Panicum coloratum*
P.deu	*Panicum deustum*
P.dic	*Panicum dichotomiflorum*
P.mid	*Panicum milioides*
P.mic	*panicum miliaceum*
P.mol	*Panicum monticola*
P.phr	*Panicum phragmitoides*
P.vir	*Panicum virgatum*
P.car	*Phaseolus carteri*
P.coc	*Phaseolus coccineus*
P.lun	*Phaseolus lunatus*
P.vul	*Phaseolus vulgaris*
P.pal	*Poa palustris*
P.dis	*Puccinellia distans*

P.lem	*Puccinellia lemmonii*
P.mar	*Puccinellia maritima*
P.nut	*Puccinellia nuttalliana*
P.mon	*Pueraria montana*
S.cer	*Secale cereale*
S.vir	*Setaria viridis*
S.ste	*Sphenostylis stenocarpa*
S.ole	*Spinacia oleracea*
S.lax	*Steinchisma laxum*
T.dic	*Triticum dicoccon*
T.mon	*Triticum monococcum*
T.tim	*Triticum timopheevii*
T.can	*Tephrosia candida*
T.pur	*Tephrosia purpurea*
T.rho	*Tephrosia rhodesica*
xTrit	*Secale cereale x Triticum aestivum*
T.aes	*Triticum aestivum*
T.aeg	*Triticum monococcum subsp. aegilopoides*
U.mos	*Urochloa mosambicensis*
U.pan	*Urochloa panicoides*
Z.may	*Zea mays*
O.sppA*	*Oryza* spp. Average Group A
A.sppB*	*Aegilops* Spp. Average Group B
L.sppC*	*Limonium* Spp. Average Group C
L.sppD*	*Limonium* Spp. Average Group D
L.sppE*	*Limonium* Spp. Average Group E

* Means have been taken between species which share identical *rbcL* sequences. *Oryza* spp. Average Group A include *O. longistaminata*, *O. glaberrima*, *O. sativa* f. *spontanea*, *O. sativa* subpsp. *indica* and *O. glumipatula*; *Aegilops* spp. Average Group B include *A. triuncialis*, *A. uniaristata*, *A. tauschii*, *A. comosa*, *A. biuncialis*, *A. cylindrica*; *Limonium* spp. Average Group C include *L. antonii-llorensii*, *L. gibertii*, *L. biflorum*; *Limonium* spp. Average Group D include *L. echioides*, *L. barceloi*, *L. balearicum*, *L. companyonis*; and *Limonium* spp. Average Group E include *L. ejulabilis*, *L. retusum*, *L. leonardi-llorensii*, *L. magallufianum*, *L. grosii*.
